# Interruption of KLF5 acetylation promotes *PTEN*-deficient prostate cancer progression by reprogramming cancer-associated fibroblasts

**DOI:** 10.1172/JCI175949

**Published:** 2024-05-23

**Authors:** Baotong Zhang, Mingcheng Liu, Fengyi Mai, Xiawei Li, Wenzhou Wang, Qingqing Huang, Xiancai Du, Weijian Ding, Yixiang Li, Benjamin G. Barwick, Jianping Jenny Ni, Adeboye O. Osunkoya, Yuanli Chen, Wei Zhou, Siyuan Xia, Jin-Tang Dong

**Affiliations:** 1 Department of Human Cell Biology and Genetics, Southern University of Science and Technology, School of Medicine, Shenzhen, Guangdong, China.; 2 Department of Hematology and Medical Oncology, Emory University School of Medicine, Atlanta, Georgia, USA.; 3 Winship Cancer Institute, Emory University, Atlanta, Georgia, USA.; 4 Inner Mongolia Institute of Quality and Standardization, Inner Mongolia Administration for Market Regulation, Hohhot, China.; 5 Departments of Pathology and Urology, Emory University School of Medicine, Atlanta, Georgia, USA.; 6 Key Laboratory of Major Metabolic Diseases and Nutritional Regulation of Anhui Department of Education, School of Food and Biological Engineering, Hefei University of Technology, Hefei, Anhui, China.

**Keywords:** Oncology, Prostate cancer

## Abstract

Inactivation of phosphatase and tensin homolog (*PTEN*) is prevalent in human prostate cancer and causes high-grade adenocarcinoma with a long latency. Cancer-associated fibroblasts (CAFs) play a pivotal role in tumor progression, but it remains elusive whether and how *PTEN*-deficient prostate cancers reprogram CAFs to overcome the barriers for tumor progression. Here, we report that *PTEN* deficiency induced Krüppel-like factor 5 (KLF5) acetylation and that interruption of KLF5 acetylation orchestrated intricate interactions between cancer cells and CAFs that enhance FGF receptor 1 (FGFR1) signaling and promote tumor growth. Deacetylated KLF5 promoted tumor cells to secrete TNF-α, which stimulated inflammatory CAFs to release FGF9. CX3CR1 inhibition blocked FGFR1 activation triggered by FGF9 and sensitized *PTEN*-deficient prostate cancer to the AKT inhibitor capivasertib. This study reveals the role of KLF5 acetylation in reprogramming CAFs and provides a rationale for combined therapies using inhibitors of AKT and CX3CR1.

## Abstract 2

KLF5 acetylation has a role in reprogramming cancer-associated fibroblasts and contributes to the synergism between AKT and CX3CR1 inhibitors in a prostate cancer model.

## Graphical abstract

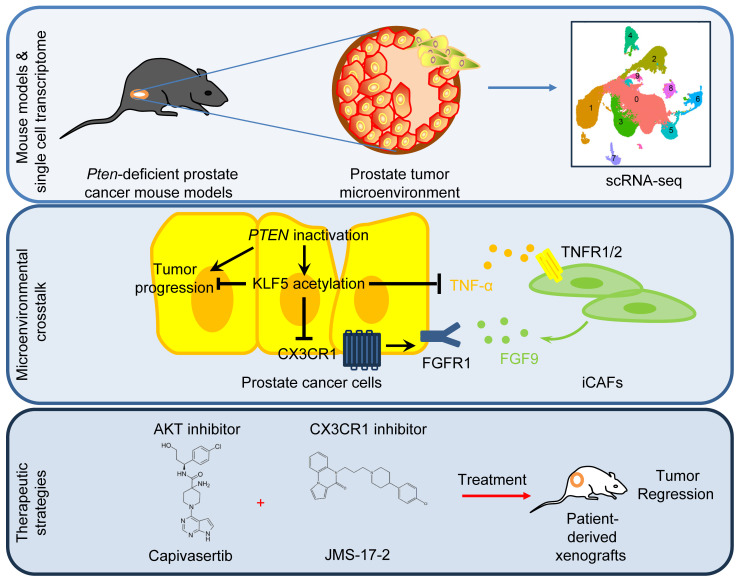

## Introduction

Prostate cancer is the most common cancer and the second leading cause of cancer-related death in men in the United States (1). Most prostate cancers are localized and androgen dependent at diagnosis and can thus be effectively treated with chemical castration, surgery, and radiation (2). Approximately 12% of prostate cancers progress to metastatic castration-resistant prostate cancer (mCRPC) (3), which contributes to mortality. Genetic drivers of prostate cancer have been extensively studied and defined to categorize disease subtypes and develop subtype-specific therapeutic strategies. One of the most potent genetic drivers of prostate cancer is phosphatase and tensin homolog (*PTEN*), a tumor suppressor gene that is mutated in approximately 20% of primary prostate cancers and in up to 50% of patients with mCRPC (4, 5).

*PTEN* inactivation results in prostate intraepithelial neoplasia (PIN) by activating PI3K/AKT signaling in genetically engineered mouse models, in which prostate cancer has a long latency to progress to high-grade adenocarcinoma, with metastasis occurring rarely (6–8). The limited tumor progression induced by *PTEN* deficiency suggests that additional molecular and cellular responses are activated to constrain tumor progression. In line with the higher frequency of *PTEN* mutations in patients with mCRPC, *PTEN* inactivation also co-occurs with other mutations in advanced prostate cancer (9). More directly, loss of *p53* or *Smad4* largely enhances the progression of prostate cancer and contributes to metastatic prostate cancer by overcoming senescence-induced by *Pten* deletion (7, 10). Activation of kinase pathways such as RAS/MAPK or HER2 also promotes tumor progression of *PTEN*-deficient prostate cancer (11, 12). On the other hand, tumor progression is not a monologue but rather an interplay with the surrounding cells in the tumor microenvironment (TME). It remains elusive whether and how TME remodeling is required for *PTEN*-deficient prostate cancer to overcome the progression barriers. Understanding these second hits for the progression of *PTEN*-deficient prostate cancer will provide a rationale for combined therapeutic strategies in the treatment of prostate cancer.

TGF-β signaling is prominent in *PTEN*-deficient prostate cancer tumors in addition to PI3K and p53 signaling (10). TGF-β/BMP-SMAD4 signaling is robustly activated in *PTEN*-null prostate cancers (10). Knockout of *Smad4*, a key component of the TGF-β pathway, results in invasive, metastatic, and lethal prostate cancers with 100% penetrance (10). TGF-β is produced by both cancer cells and the TME and actively reshapes the TME (13). While TGF-β inhibits tumor growth in early-stage tumors, it induces epithelial-mesenchymal transition (EMT) and promotes cancer metastasis in later-stage tumors (14–19).

Acetylation of the transcription factor Krüppel-like factor 5 (KLF5) at lysine 369 (K369) has been identified as a posttranscriptional modification downstream of TGF-β. KLF5 acetylation is induced by TGF-β via the SMAD-recruited p300 acetylase (20, 21). Acetylated KLF5 (Ac-KLF5) then forms a transcriptional complex, distinct from that of deacetylated KLF5 (deAc-KLF5), which is essential for TGF-β to function in gene regulation, cell proliferation, and tumorigenesis (20–23). However, it remains unclear whether and how KLF5 acetylation remodels the TME in prostate cancer progression. In our most recent study, we found that Ac-Klf5 is essential for proper basal-to-luminal differentiation in the prostate and that loss of Klf5 acetylation in basal progenitor cells results in low-grade PIN (24), suggesting a role of Klf5 acetylation in prostate cancer progression. More important, we established a genetically engineered mouse model (GEMM) to conditionally interrupt Klf5 acetylation, providing a unique animal model to address the role of Ac-KLF5 in the progression of *PTEN*-deficient prostate cancer (24).

Here, we found that Klf5 acetylation at K358 (a homologous site of human KLF5 K369) was significantly (*P* < 0.001) increased by *Pten* loss in mouse prostates and phosphorylated AKT (p-AKT) activation in human prostates. Interruption of Klf5 acetylation promoted tumor growth in *Pten*-deficient prostate cancer, as indicated by larger tumor sizes and enhanced cell proliferation. Mechanistically, the KLF5 acetylation–dependent barrier induced by *PTEN* deficiency constrained prostate tumor growth by attenuating FGF receptor 1 (FGFR1) signaling. Deacetylation of KLF5 in prostate cancer cells stimulated inflammatory cancer-associated fibroblasts (iCAFs) through TNF-α to release FGF9, which in turn activated FGFR1 signaling in prostate cancer cells. In addition to the paracrine signaling, deAc-KLF5 induced CX3CR1, which was required by FGF9 to activate FGF receptor 1 (FGFR1) signaling. Inhibition of CX3CR1 sensitized *PTEN*-deficient prostate cancer to the AKT inhibitor capivasertib. This study not only clarifies the role of KLF5 acetylation in reciprocal communications between prostate cancer cells and iCAFs in *PTEN*-deficient tumors, but also provides a proof of concept for posttranslational modifications (PTMs) as essential molecular events induced by *PTEN* inactivation to stall prostate cancer progression.

## Results

### PTEN deficiency induces KLF5 acetylation in mouse and human prostate tumors.

**Figure 1:**
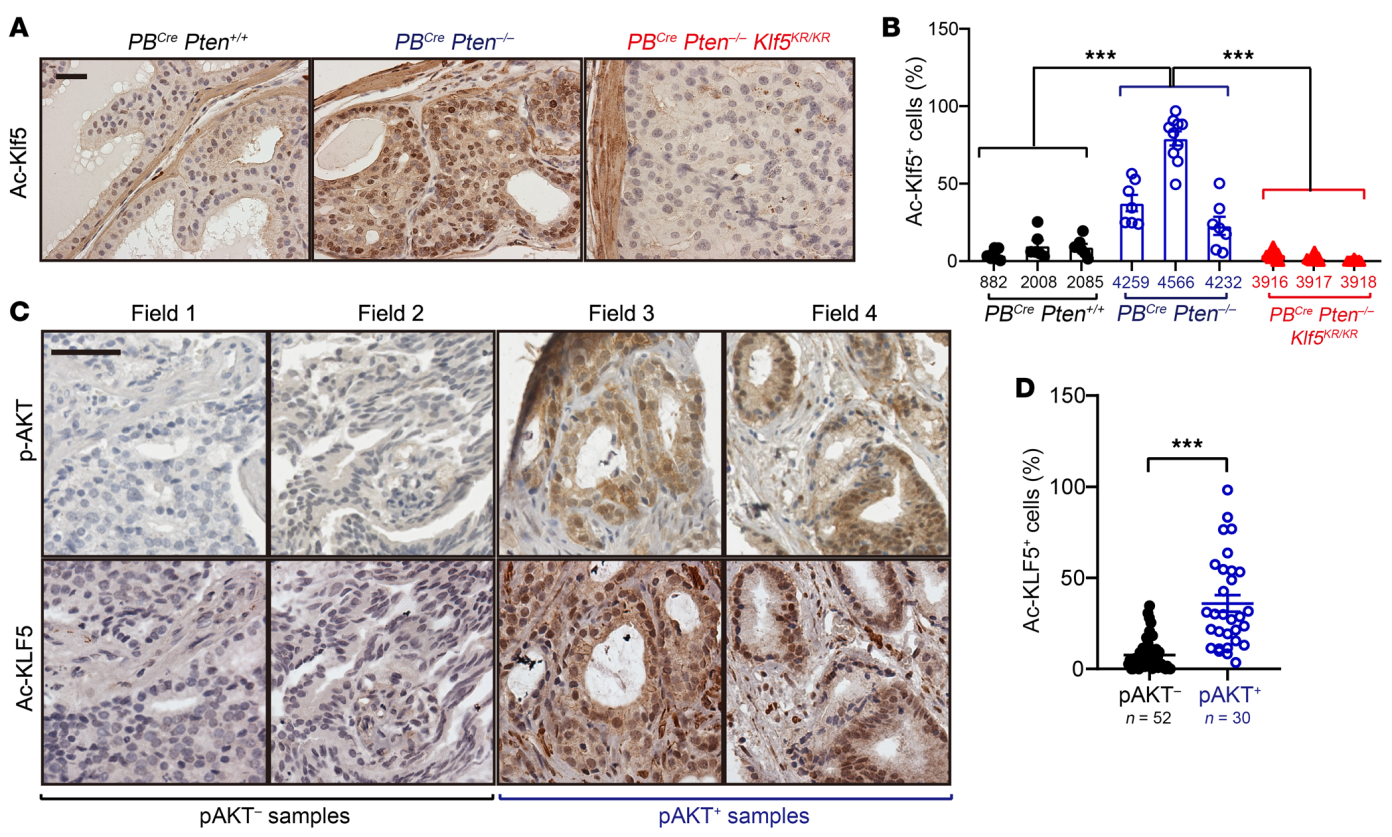
*PTEN* loss induces KLF5 acetylation in mouse and human prostates. (**A** and **B**) IHC staining of acetylated Klf5 at K358 in 4-month-old mice with the indicated genotypes, as shown in the representative images (**A**) and statistical analysis (**B**). ***P < 0.001, by 2-way ANOVA. (**C** and **D**) IHC staining of acetylated KLF5 at K369 in human prostate cancer specimens with or without AKT activation, as indicated by the representative images (**C**) and statistical analysis (**D**). Scale bars: 50 μm (**A** and **C**). Data are shown as the mean ± SEM. ***P < 0.001, by 2-tailed Student’s *t* test.

KLF5 acetylation at K369 is induced by TGF-β and has been identified as a crucial PTM downstream of TGF-β in mediating TGF-β’s functions (20, 21). Given the robust activation of TGF-β in *PTEN*-deficient prostate cancer, we tested whether KLF5 acetylation at K369 is affected by PTEN/PI3K/p-AKT signaling. Prostate-specific *Pten* knockout led to adenocarcinoma in mouse prostate (6) and induced Klf5 acetylation at K358 (a homologous site of human KLF5 K369 Figure 1, A and B), as indicated by IHC staining. Knockin of the *Klf5^K358R^* (*Klf5^KR^*) mutant in *Pten*-null mouse prostate successfully depleted Klf5 acetylation, validating the induction of Klf5 acetylation at K358 by *Pten* knockout (Figure 1, A and B).

*PTEN* loss activated PI3K/AKT signaling to promote prostate cancer progression. In human prostate cancer samples, we found that Ac-KLF5 expression was significantly higher when AKT was activated (Figure 1, C and D), consistent with the findings in the GEMM. We also evaluated the expression levels of total KLF5 in both GEMM and human prostate cancer specimens but did not observe significant differences in tissues with or without AKT activation (Supplemental Figure 1, A and B; supplemental material available online with this article; https://doi.org/10.1172/JCI175949DS1).

### Interruption of Klf5 acetylation by the K358R mutation promotes Pten-null prostate tumor growth.

**Figure 2:**
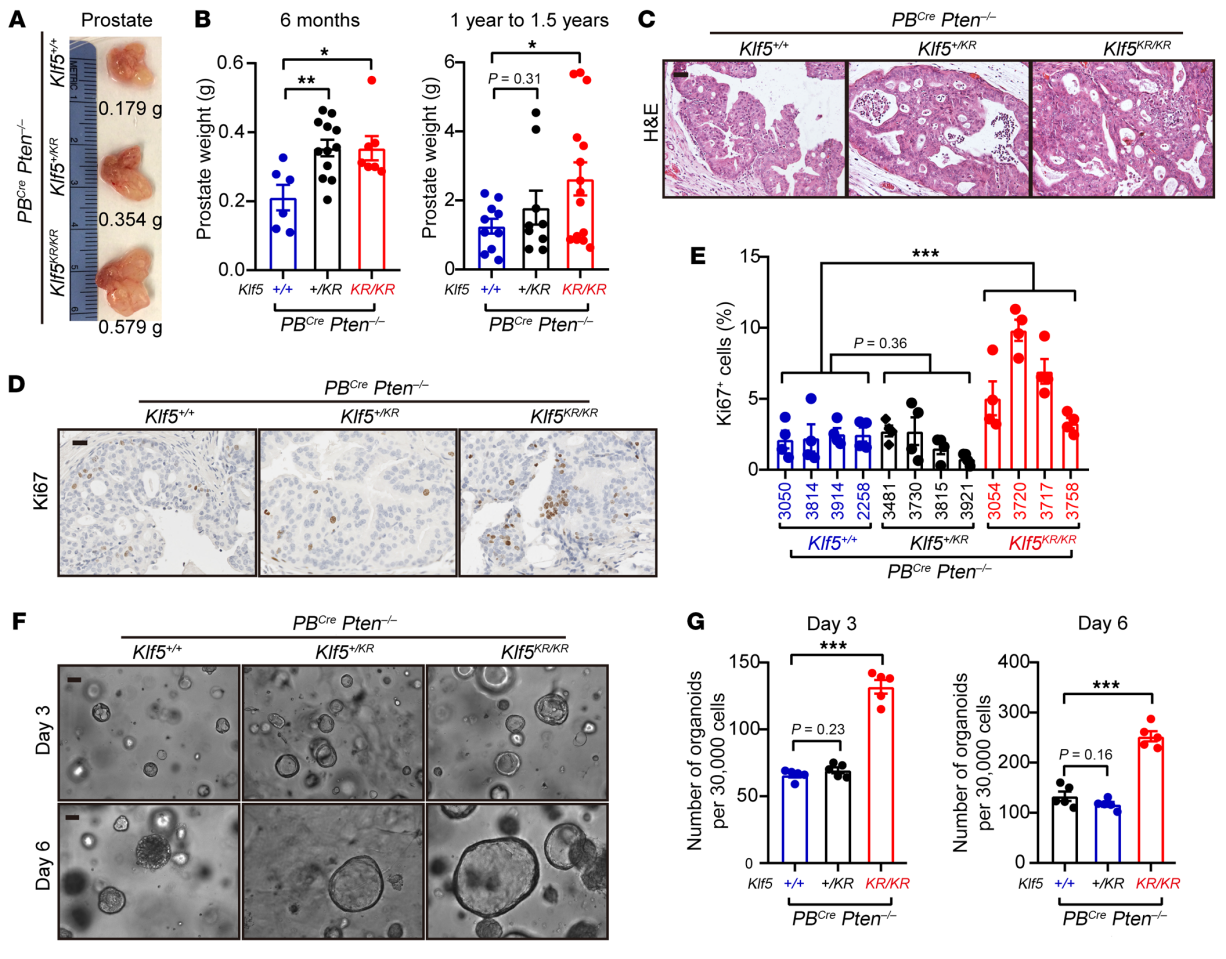
Deacetylation of Klf5 accelerates cell proliferation and the growth of tumors induced by *Pten* loss in the prostate. (**A** and **B**) Knockin of *Klf5^K358R^* (*Klf5^KR^*) increased the weight of *Pten*-deficient mouse prostates, as indicated by the tumor images (**A**) and tumor weights (**B**). (**C**–**E**) Histological features of 16-week mouse prostates revealed by H&E staining (**C**) and proliferation index detected by Ki67 IHC staining (**D** and **E**). (**F** and **G**) Organoid culture of prostate epithelial cells with the indicated genotypes, as indicated by representative organoid images (**F**) and statistical analysis of organoid numbers (**G**). Scale bars: 50 μm. Data are shown as the mean ± SEM. *P < 0.05, **P < 0.01, and ***P < 0.001, by 2-tailed Student’s *t* test (**B** and **G**) and 2-way ANOVA (**E**).

Knockin of the *Klf5^KR^* mutant successfully interrupted Klf5 acetylation in *Pten*-deficient mouse prostates (Figure 1, A and B), providing an ideal model with which to test how Klf5 acetylation affects *Pten*-deficient prostate cancer. *Klf5^KR^* knockin led to larger tumors in *Pten*-deficient prostates of 6-month-old mice, as indicated by the tumor images and prostate weights (Figure 2, A and B). In addition, knockin of 1 allele of *Klf5^KR^* appeared to efficiently enlarge tumor sizes within 6 months, although the increase in tumor sizes did not reach significance at 1 to approximately 1.5 years, probably due to the considerable variations among prostate weights (Figure 2B). Further pathological evaluation indicated that knockin of *Klf5^KR^* resulted in more proliferative cells in prostate tumors, as suggested by both the mitotic images and frequency of Ki67^+^ cells (Figure 2, C–E), but did not significantly altered the expression patterns of epithelial markers, such as Ar, Ck5, and Ck8 (Supplemental Figure 1C). Mouse prostate cancer cells were used for organoid formation assays (Figure 2, F and G). *Klf5^KR^* knockin gave rise to more and larger organoids, indicating a role of deAc-KLF5 in promoting prostate tumor growth. One allele of *Klf5^KR^* knockin appeared insufficient to promote organoid formation (Figure 2, F and G), implying that the extent of Klf5 acetylation may be an essential factor in suppressing tumor growth. Collectively, interruption of Klf5 acetylation at K358 promoted prostatic tumor growth by accelerating cell proliferation.

### Deacetylation of KLF5 causes hyperactivated FGFR1 signaling in PTEN-deficient tumors.

**Figure 3:**
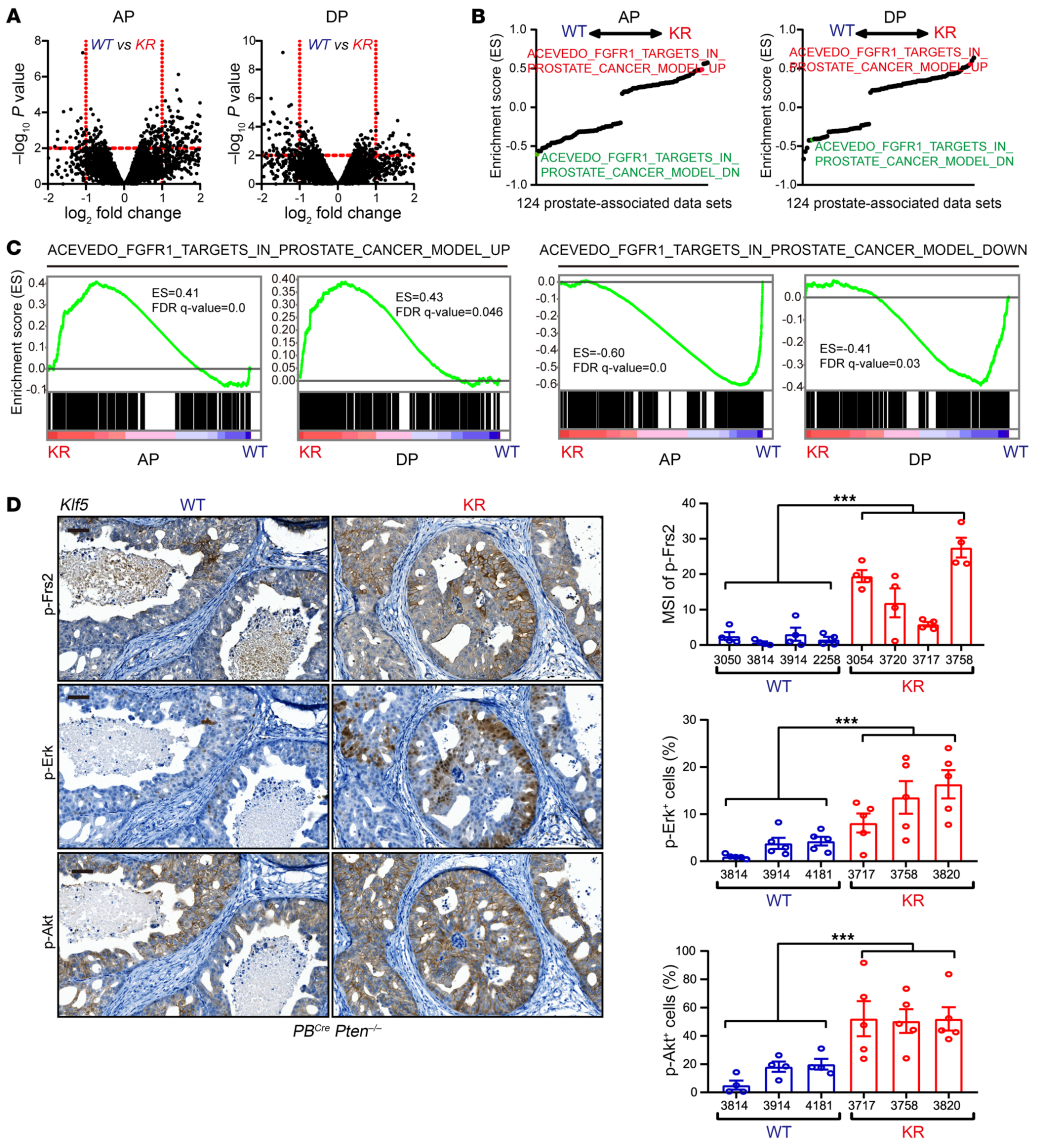
Interruption of Klf5 acetylation enhances FGFR1 signaling in *Pten*-deficient prostate tumors. (**A**) Differential gene expression caused by *Klf5^K358R^* (KR) knockin in *Pten*-loss mouse prostates, as determined by RNA-Seq in APs and dorsal DPs. (**B**) GSEA of RNA-Seq data on prostates from 16-week-old *PB^Cre^* *Pten^–/–^* *Klf5^KR/KR^* (KR) and *PB^Cre^* *Pten^–/–^* *Klf5^+/+^* (WT) mice from 124 prostate-associated data sets. (**C**) GSEA using the gene sets containing FGFR1 upregulated and downregulated genes from Acevedo et al. (42). (**D**) Knockin of *Klf5^KR^* enhances the activation of Erk, Akt, and Frs2, as detected by IHC staining for p-Erk^Thr202/Tyr204^, p-Akt^Ser473^, and p-Frs2^Tyr436^. Scale bars: 50 μm. MSI, mean staining intensity. Data are shown as the mean ± SEM. ***P < 0.001, by 2-way ANOVA.

To understand the underlying mechanisms by which deacetylation of Klf5 promotes *Pten*-deficient prostate cancer progression, we performed RNA-Seq to identify differentially expressed genes (DEGs) in *Pten*-null mouse prostates with or without *Klf5^KR^* knockin. Anterior and dorsal prostates were dissected for RNA-Seq separately to capture gene expression (Figure 3A and Supplemental Data Sets 1 and 2). In anterior prostates (APs), *Klf5^KR^* knockin induced the expression of 31 genes and suppressed the expression of 162 genes (fold change >2 and *P* < 0.01). In dorsal prostates (DPs), *Klf5^KR^* knockin induced the expression of 107 genes and suppressed the expression of 80 genes (fold change >2 and *P* < 0.01). Functional annotations of differential gene expression by Gene Ontology (GO) analysis revealed the top 20 significant (adjusted *P* < 0.05) biological processes in both APs and DPs (Supplemental Figure 2, A and B). Notably, genes regulating cell-cell adhesion were enriched in both APs and DPs (Supplemental Figure 2, A and B). Further investigation of the genes associated with the top enriched biological processes suggested that *Klf5^KR^* knockin enriched several genes involved in cell-cell communications, specifically some cytokines and cytokine receptors (Supplemental Figure 2C). Given that Smad4 is induced by *Pten* knockout and constrains tumor progression (10), we compared the DEGs after *Klf5^KR^* knockin with those caused by *Smad4* knockout. The genes that are upregulated by *Smad4* knockout were enriched in *Klf5^KR^*-knockin–upregulated genes, and the *Smad4*-knockout–downregulated genes were enriched in *Klf5^KR^*-knockin–suppressed genes (Supplemental Figure 2D). These findings suggest that Klf5 acetylation is a barrier for *Pten*-null prostate cancer progression, just like Smad4 (10).

Focusing on the gene profiles altered by the interruption of Klf5 acetylation, we further performed gene set enrichment analysis (GSEA) using a gene set library containing 124 prostate-associated gene sets from the Molecular Signatures Database (MSigDB). Interestingly, FGFR1-regulated gene sets were among the top enriched sets in both AP and DP (Figure 3B). FGFR1-induced genes were significantly enriched among *Klf5^KR^*-knockin–upregulated genes, and FGFR1-downregulated genes were significantly enriched in *Klf5^KR^*-knockin–suppressed genes (Figure 3C). The enrichment was significant in both AP and DP (Figure 3C). These GSEA data clearly indicate that interruption of Klf5 acetylation at K358 further enhanced FGFR1 signaling in *Pten*-deficient prostate tumors.

We also confirmed the activation of Fgfr1 signaling in *Pten*-deficient mouse prostates with *Klf5^KR^* knockin by detecting p-Frs2, p-Erk, and p-Akt, the canonical downstream signals of Fgfr1 (25). As expected, interruption of Klf5 acetylation at K358 significantly induced the activation of Frs2, Erk, and Akt (Figure 3D), indicating that the acetylation of Klf5 at K358 constrained Fgfr1 activation in *Pten*-knockout mouse prostates. The activation of Fgfr1 by *Klf5^KR^* knockin was also confirmed by Western blotting (Supplemental Figure 1D), and consistent results were achieved.

### Single-cell RNA-Seq reveals enhanced FGF signaling from fibroblasts to cancer cells.

**Figure 4:**
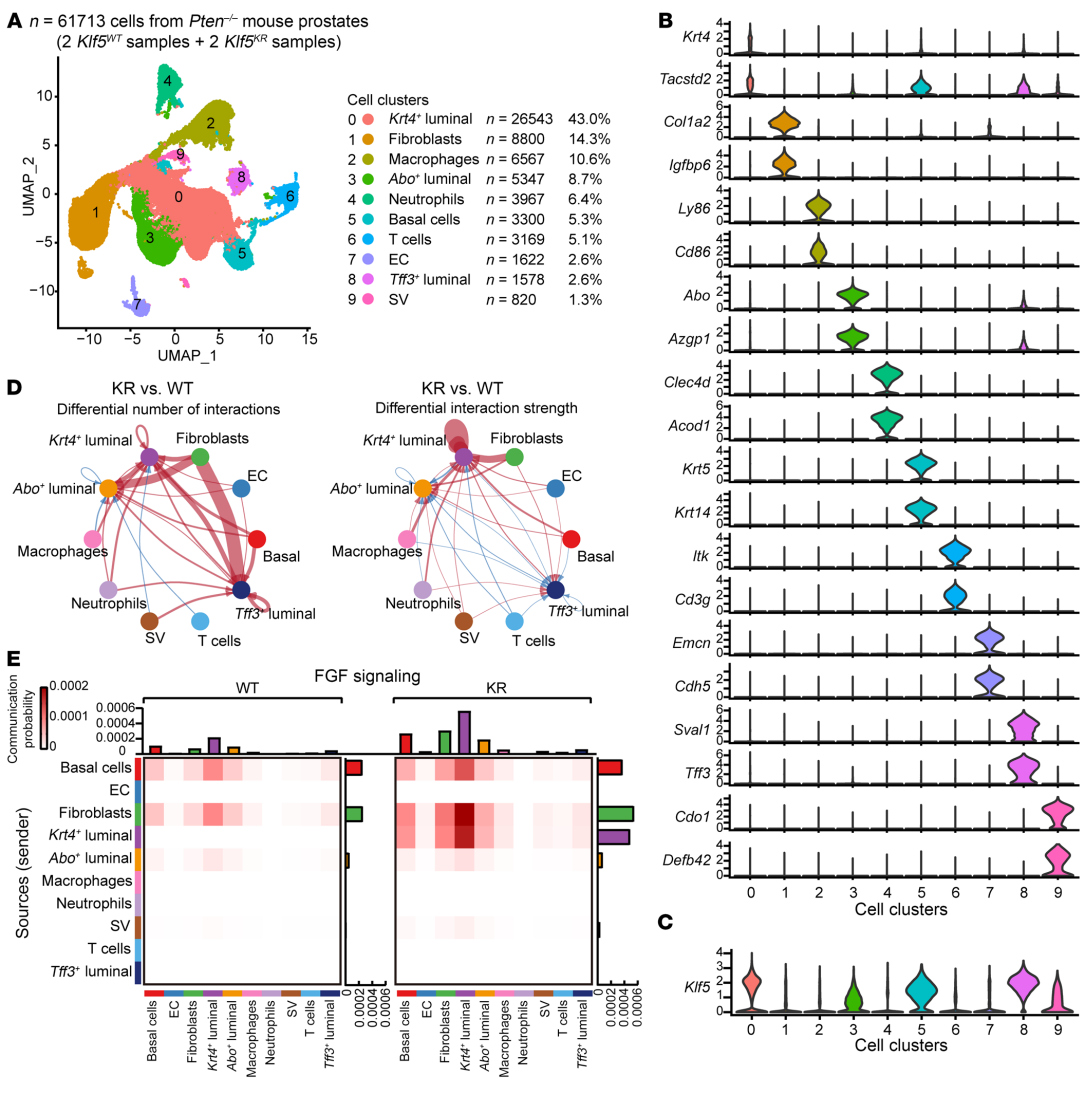
Single-cell transcriptomics analysis reveals enhanced FGF signaling from fibroblasts to cancer cells after the interruption of Klf5 acetylation. (**A**) Visualization of the annotated clusters of 61,713 single cells from *Pten^–/–^* mouse prostates (*n* = 2 mice for each genotype) based on the expression of known marker genes by uniform manifold approximation and projection (UMAP) (left panel). The numbers and percentages of the assigned cell types are summarized in the right panel. (**B** and **C**) Violin plots showing the expression levels of representative marker genes (**B**) and *Klf5* (**C**) across the main clusters (*n* = 61,713 cells). (**D**) Differential number (left) and strength (right) of interactions from the main clusters to the 3 luminal clusters between *PB^Cre^* *Pten^–/–^* *Klf5^KR/KR^* (KR) and *PB^Cre^* *Pten^–/–^* *Klf5^+/+^* (WT) mouse prostates, as identified by CellChat. The red lines represent activated interactions, and the blue lines represent suppressed interactions in the KR group. Thicker lines indicate greater changes in interactions. (**E**) The communication probability of FGF signaling was calculated by CellChat and is shown as a heatmap. EC, endothelial cell; SV, seminal vesicle epithelial cell.

To investigate whether and how TME signaling is attributed to FGFR1 overactivation, we performed single-cell RNA-Seq (scRNA-Seq) to analyze the crosstalk between prostate cancer cells and other types of cells in the microenvironment. We profiled 61,713 individual cells from fresh, dissociated whole prostates of four 16-week-old *PB^Cre^* *Pten^–/–^* mice after quality control. These cells included 14,464 and 18,024 cells from two *Klf5^WT^* (WT) mice, and 12,310 and 16,915 cells from two *Klf5^KR^* (KR) mice. Clustering analysis identified 10 distinct clusters of 820 to 26,543 cells each (Figure 4A). Cells from the 4 mouse prostates were distributed evenly in all 10 clusters, and each cluster contained cells from all the 4 mice (Supplemental Figure 3A).

To annotate the cell clusters, we performed differential gene expression analysis through which we successfully identified distinct marker genes for each cluster. (Figure 4B, Supplemental Figure 3B, and Supplemental Data Set 3). We took into account that the cells analyzed in our scRNA-Seq assay contained various cell components, including normal mouse prostate cells, prostate cancer cells, and other microenvironmental cells. Therefore, we used marker genes from Guo et al. for the cell-type identification of the normal mouse prostates (26) and those from Chan et al. for the cell-type identification of mouse prostate cancer tissues (27). The identities of cell clusters were further validated by marker genes in PanglaoDB (28). In most clusters, typical cell lineage–specific markers were found on the top of the marker gene list (Supplemental Figure 3B), and 2 representative markers are shown in Figure 4B. Canonical luminal cells markers (e.g., *Krt8* and *Krt18*) were found in clusters 0, 3, and 8 (Supplemental Figure 3C), and these clusters were subsequently distinguished on the basis of their characteristic gene expression. In comparison with previous studies, the *Abo*^+^ luminal cluster demonstrated striking similarity to the luminal A cells, which are a cluster of cells identified in normal prostates (26). The *Krt4*^+^ luminal cells shared marker genes that align with adenocarcinoma cells with luminal phenotypes (27). Remarkably, the *Tff3*^+^ luminal cluster consistently expressed *Tff3*, *Sval1*, *Agr2*, and *Ffar4*, which are the primary marker genes highlighted in *Tff3*^+^ clusters by Chan et al. (27).

Plotting the cell clusters with Klf5 expression, we found that most of them were epithelial cells (Figure 4C), consistent with the previous concept that Klf5 is an epithelial factor (29, 30). Notably, the Cre activity of these *PB^Cre/+^* mice was specific to the epithelial cells of mouse prostates (31). Considering this specificity, we used inferCNV to assess the effect of oncogenic signaling on various epithelial cell types. Interestingly, the *Krt4^+^* luminal cells had the highest number of copy number variations (CNVs) (Supplemental Figure 3D), suggesting the presence of cancer-like characteristics of this cell cluster.

We analyzed the cell-cell communications in the TME and found that the disruption of Klf5 acetylation in *Pten*-deficient tumors resulted in the most substantial changes in interaction strength within *Krt4*^+^ luminal cells, fibroblasts, and macrophages (Supplemental Figure 3E). Putting the luminal cells as the signaling receiver, fibroblasts were the primary sources of signaling activation subsequent to *Klf5^KR^* knockin (Figure 4D). Dissecting the specific signaling pathways revealed that FGF was one of the top signaling pathways that was boosted by deAcKlf5 (Supplemental Figure 3F). Strikingly, when we focused on FGF signaling, we found that the *Krt4*^+^ luminal clusters received the highest FGF signaling after *Klf5^KR^* knockin and that the primary source was from fibroblasts (Figure 4E).

### Increased FGF9 release in CAFs activates FGFR1 signaling in tumor cells with Klf5^KR^ knockin.

**Figure 5:**
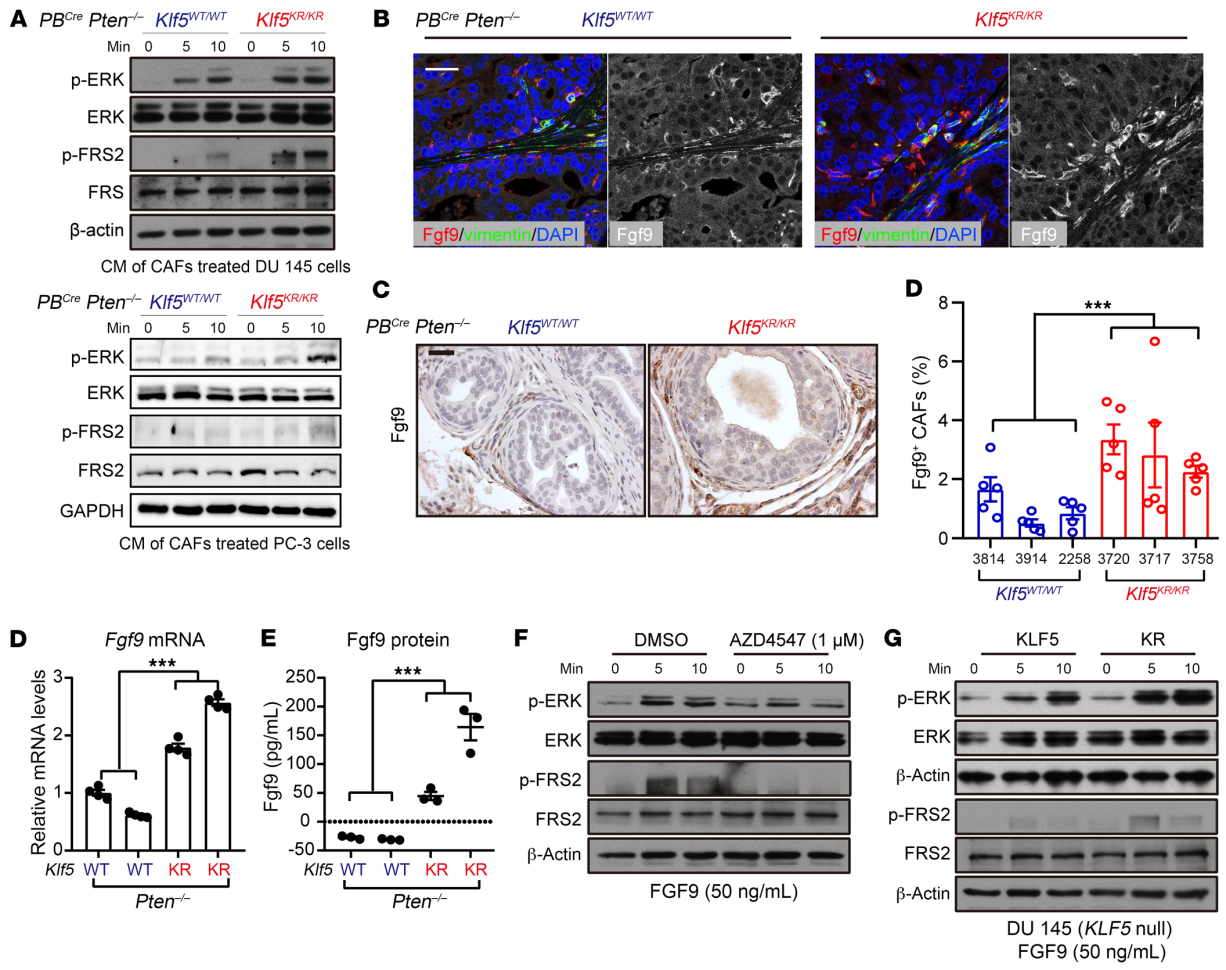
Increased Fgf9 in CAFs contributes to hyperactivated FGFR1 signaling in *Klf5^KR^* tumor cells with the *Klf5^KR^* knockin. (**A**) CM containing CAFs from *PB^Cre^* *Pten^–/–^* *Klf5^KR/KR^* mice were more potent in activating FGFR1 in DU 145 and PC-3 prostate cancer cells, as indicated by the expression levels of p-ERK^Thr202/Tyr204^ and p-FRS2^Tyr436^ detected by Western blotting. (**B** and **C**) Fgf9 expression levels in *Pten*-null mouse prostates with the indicated *Klf5* statuses, as measured by IF staining (**B**) and IHC staining (**C**). The mice used were 16 weeks of age. Scale bars: 50 μm. (**D** and **E**) Fgf9 mRNA and protein expression levels in isolated CAFs from mice of the indicated genotypes, as determined by real-time qPCR (**D**) and ELISA (**E**). WT is *PB^Cre^* *Pten^–/–^* *Klf5^WT/WT^* and KR is *PB^Cre^* *Pten^–/–^* *Klf5^KR/KR^*. ***P < 0.001, by 2-way ANOVA (**C**–**E**). Data are shown as the mean ± SEM. (**F**) FGF9-induced FGFR1 activation was suppressed by the FGFR1 inhibitor AZD4547. (**G**) FGF9 was more potent in activating FGFR1 signaling, as indicated by the expression levels of p-ERK^Thr202/Tyr204^ and p-FRS2^Tyr436^ by Western blotting. In **F** and **G**, DU 145 cells were treated as indicated in the figures.

To further validate the observation that the microenvironmental signaling from fibroblasts is attributable to FGFR1 overactivation, we collected conditioned media (CM) from CAFs derived from *Pten*-deficient mouse prostates. We found that these CM were capable of inducing FGFR1 activation in prostate cancer cells, as indicated by the phosphorylation of ERK and FGF receptor substrate 2 (FRS2) (Figure 5A). Moreover, the CM from *Klf5^KR^*-knockin mice were more potent than the CM counterpart (Figure 5A), suggesting that more cytokines that activate FGFR1 signaling could be released by CAFs from *Klf5^KR^*-knockin mice.

In the scRNA-Seq data, we found that most FGFs were released by fibroblasts and that *Fgf2*, *Fgf7*, *Fgf9*, *Fgf10*, and *Fgf18* were the top differential *Fgfs* that were upregulated in the fibroblasts of *Klf5^KR^* prostates (Supplemental Figure 4A). Further investigation of the expressed Fgfs in RNA-Seq data revealed that *Fgf9* was the only Fgf that was significantly induced by the *Klf5^KR^* mutant in *Pten*-deficient mouse prostates (Supplemental Figure 4B). Focusing on the overlapped Fgf, Fgf9, we confirmed the increased expression levels of Fgf9 by immunofluorescence (IF) (Figure 5B) and IHC staining (Figure 5C). Consistent with the scRNA-Seq data, our IF and IHC staining data confirmed that the Fgf9 signal mainly occurred in CAFs (Figure 5, B and C). We further isolated CAFs from *Pten*-deficient mouse prostates and validated the increase in Fgf9 expression in the CAFs from *Klf5^KR^*-knockin prostates, as indicated by both real-time quantitative PCR (qPCR) and ELISA (Figure 5, D and E).

FGF9 activated FGFR1 signaling in a dose-dependent manner within 15 minutes in DU 145 prostate cancer cells (Supplemental Figure 4, C and D), as indicated by the phosphorylation of ERK and FRS2. This activation was eliminated by the FGFR1 inhibitor AZD4547 or by knockdown of FGFR1 (Figure 5F and Supplemental Figure 4E). We also tested the activation effects of FGF9 on FGFR1 signaling in prostate cancer cells with or without the *KLF5^KR^* mutant. Interestingly, FGFR1 signaling in DU 145 cells with the *KLF5^KR^* mutant was more sensitive to FGF9 (Figure 5G), implying that an endogenous pathway in the tumor cells could be involved in the activation of FGFR1 signaling. Collectively, FGF9 was a ligand of FGFR1 that was mainly released by CAFs and activated FGFR1 signaling in prostate cancer. The overactivated FGFR1 signaling in Ac-Klf5–deficient and *Pten*-null prostate cancers can be attributed at least partly to the increased expression of FGF9.

### deAc-KLF5 upregulates TNF-α in cancer cells to increase FGF9 release by CAFs.

**Figure 6:**
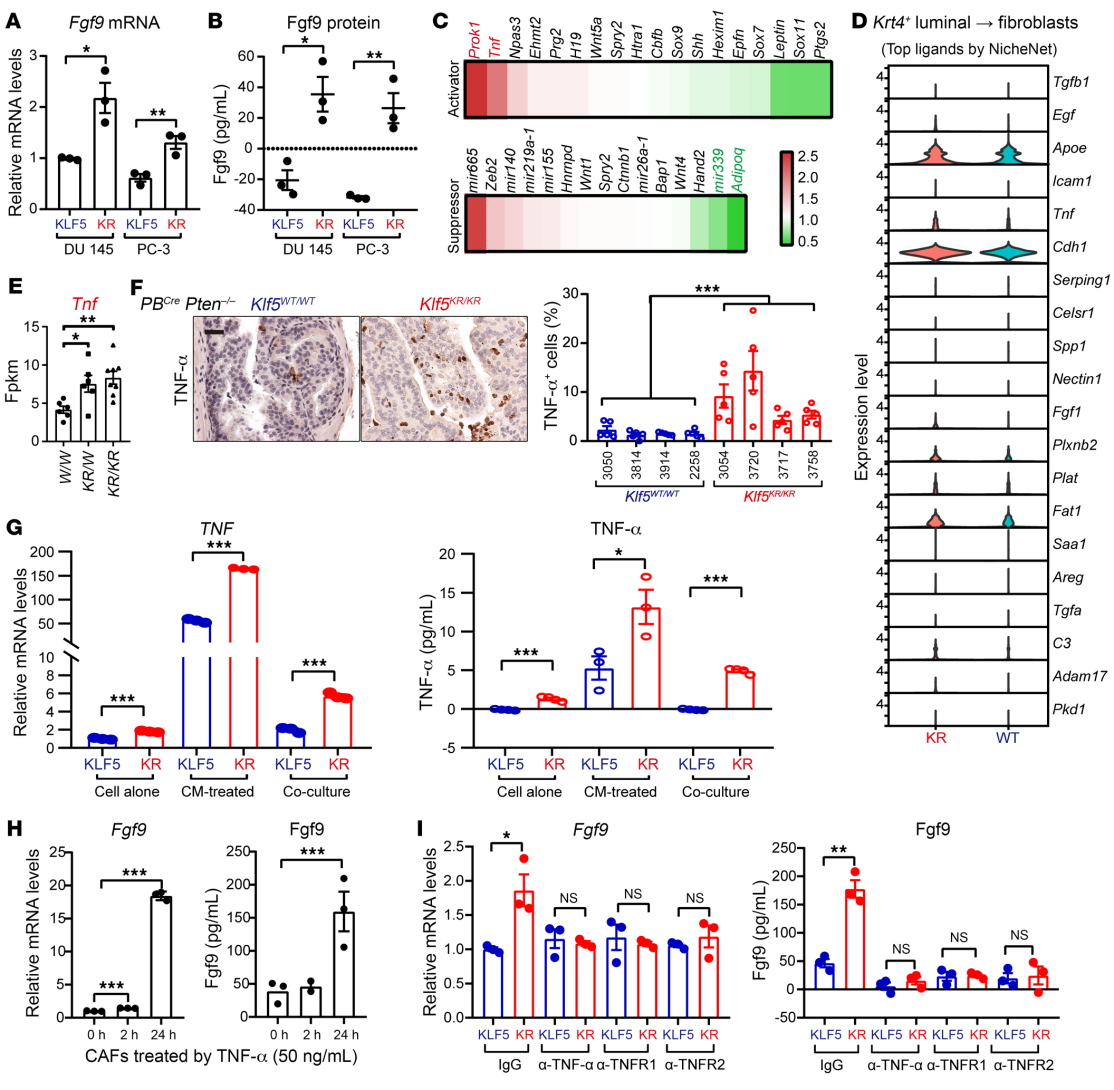
Interruption of Klf5 acetylation upregulates TNF-α in *Pten*-null tumor cells to induce FGF9 secretion in CAFs. (**A** and **B**) Expression levels of Fgf9 mRNA and protein were higher in isolated CAFs when cocultured with 2 prostate cancer cell lines PC-3 and DU 145 with the KLF5^KR^ mutant, as detected by real-time qPCR (**A**) and ELISA (**B**). (**C**) Heatmap showing expression of activators and suppressors of FGF9 as reviewed from 617 publications. Red and green indicate the genes upregulated and downregulated by the *Klf5^KR^* mutant. (**D**) The top ligands that signal fibroblasts from *Krt4*^+^ luminal cells were calculated by NicheNet, and their expression levels in *Krt4*^+^ luminal cells are shown as violin plots. (**E**) Plots of *Tnf* expression as detected by RNA-Seq. *W/W*, *PB^Cre^* *Pten^–/–^* *Klf5^WT/WT^*; *KR/W*, *PB^Cre^* *Pten^–/–^* *Klf5^WT/KR^*; *KR/KR*, *PB^Cre^* *Pten^–/–^* *Klf5^KR/KR^*. (**F**) IHC staining for Tnf-α in mouse prostate tumors of the indicated genotypes. Scale bar: 50 μm. (**G**) The expression levels of TNF-α mRNA and protein were higher in DU 145 cells expressing the *KLF5^KR^* mutant, as indicated by real-time qPCR (left) and ELISA (right). DU 145 cells were cultured under the indicated conditions. CAFs from *Pten*-deficient mouse prostate tumors were used to produce CM and cocultured with DU 145 cells. (**H**) TNF-α induced Fgf9 expression levels in CAFs, as indicated by real-time qPCR (left) and ELISA (right). (**I**) Blockage of TNF-α signaling by the neutralizing antibodies against TNF-α (5 ng/mL), TNFR1 (20 μg/mL), or TNFR2 (5 ng/mL) suppressed Fgf9 expression that was induced in CAFs by expression of the *KLF5^KR^* mutant in DU 145 cells. Data are shown as the mean ± SEM. *P < 0.05, **P < 0.01, and ***P < 0.001, by 2-tailed Student’s *t* test (**A**, **B**, **E**, and **G**–**I**) and 2-way ANOVA (**F**).

Because *PB^Cre^* contains a probasin promoter and only directs Cre-mediated recombination in epithelial cells of the prostate (31), we asked whether the enhanced secretion of Fgf9 in CAFs is attributable to stimulus from epithelial cells. Coculturing of CAFs with prostate cancer PC-3 and DU 145 cells with the *KLF5^KR^* mutant released more Fgf9 than did the WT control at both the mRNA and protein levels (Figure 6, A and B), indicating that the signal from prostate cancer cells was essential for CAFs to release FGF9.

A thorough literature review revealed several activators and suppressors of FGF9 (Figure 6C). Focusing on the signaling crosstalk between *Krt4*^+^ luminal cells and fibroblasts, we conducted a more in-depth analysis of the top differential ligands between the *Klf5^KR^* and *Klf5^WT^* groups within the scRNA-Seq data (Figure 6D). TNF, encoding TNF-α, was emergent as an FGF9 regulator with significant upregulation in the Klf5^KR^ group, as indicated by both the scRNA-Seq and RNA-Seq data (Figure 6, D and E). Through the estimation of signaling pathway activities, we verified the augmented activation of TNF in *Krt4*^+^ luminal clusters within the *Klf5^KR^* group (Supplemental Figure 5A). More directly, CellChat analysis of the scRNA-Seq data revealed that *Krt4*^+^ luminal cells, macrophages, and neutrophils were the 3 predominant sources of TNF signaling enhancement due to *Klf5^KR^* knockin (Supplemental Figure 5B).

We further measured TNF-α expression levels by IHC staining in the prostates of *PB^Cre^* *Pten^–/–^* *Klf5^KR/KR^* and *PB^Cre^* *Pten^–/–^* *Klf5^+/+^* mice and confirmed that *Klf5^KR^* knockin significantly induced TNF-α expression in *Pten*-deficient mouse prostate cancer (Figure 6F). A further IF staining assay indicated that the expression of TNF-α induced by *Klf5^KR^* knockin occurred in both epithelial cells and CD11b^+^ macrophages (Supplemental Figure 5C). To determine whether deAc-KLF5 affects TNF-α secretion in cancer cells, we measured the expression levels of TNF-α in DU 145 prostate cancer cells with KLF5^WT^ and KLF5^KR^ in different culture conditions, including with cancer cells alone, cancer cells treated by CAF CM, and cancer cells cocultured with CAFs (Figure 6G). As indicated by real-time qPCR and ELISA, DU 145 cells with KLF5^KR^ released more TNF-α (Figure 6G). Interestingly, the basal levels of TNF-α were increased when the cancer cells were treated with CAF CM or cocultured with CAFs (Figure 6G), suggesting a potential role of the crosstalk between cancer cells and CAFs in TNF-α secretion.

Functionally, after a 24-hour treatment, we found that TNF-α induced Fgf9 expression levels in CAFs (Figure 6H). Furthermore, in the cocultures of CAFs and DU 145 cancer cells, the blockage of TNF-α by the neutralizing antibodies against TNF-α, TNFR1, and TNFR2 effectively eliminated the increase in Fgf9 secretion by CAFs caused by *KLF5^KR^* knockin (Figure 6I). These findings indicate that deacetylation of KLF5 in cancer cells signaled CAFs to release more FGF9 in a TNF-α–dependent manner.

### Klf5 deacetylation amplifies the FGF/TNF signaling interplay between iCAFs and tumor cells.

**Figure 7:**
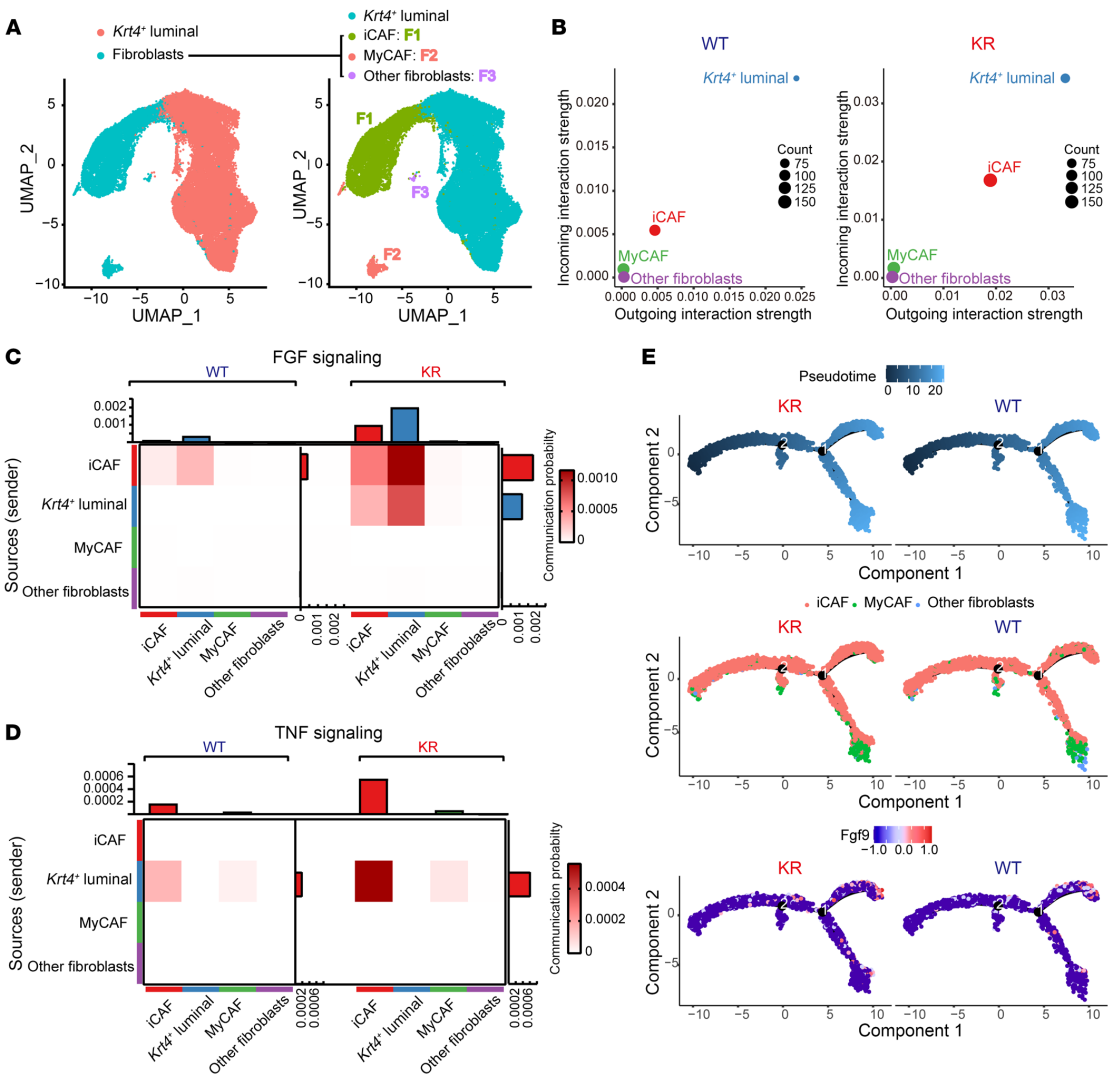
Klf5 deacetylation enhances FGF/TNF signaling crosstalk between iCAFs and prostate cancer cells. (**A**) UMAP visualization of the annotated clusters of *Krt4*^+^ luminal cell and fibroblast subsets in scRNA-Seq (*n* = 35,343 cells). Fibroblasts were further divided into iCAFs, MyCAFs, and undefined fibroblasts (other fibroblasts) on the basis of their representative marker genes. (**B**) Enhanced strength of interactions between iCAFs and *Krt4*^+^ luminal cells after Klf5 deacetylation. (**C** and **D**) The communication probability of FGF (**C**) and TNF (**D**) signaling between *Krt4*^+^ luminal cells and different fibroblast subsets was calculated by CellChat and shown as heatmaps. (**E**) CAFs were ordered along pseudotime trajectories by Monocle2, and cell types and relative Fgf9 expression levels are shown.

In our study to further understand how deacetylation of Klf5 in prostate cancer cells reprograms fibroblasts, 3 subclusters of fibroblasts were revealed by their distinct marker gene expression (Figure 7A and Supplemental Figure 6A). The 3 fibroblast clusters comprised an iCAF cluster, which expressed canonical markers like *Dpt*, *Gsn*, *Svep1*, *Plpp3*, and *Il6*; a myofibroblastic CAF (MyCAF) cluster, which exhibited marker genes such as *Col15a1*, *Tpm2*, *Tnc*, and *Cald*; and an unclassified fibroblast cluster (other fibroblasts) (32). It was evident that deAcKlf5 mainly intensified the signaling interaction between *Krt4*^+^ luminal cells and iCAFs, as revealed by CellChat analysis (Figure 7B and Supplemental Figure 6B). Moreover, the Fgf9 induced by *Klf5^KR^* knockin occurred in iCAFs, but not in other types of CAFs (Supplemental Figure 6C). As expected, *Klf5^KR^* knockin led to an augmentation of FGF and TNF signaling within the cell subsets including fibroblasts and *Krt4*^+^ luminal cells (Supplemental Figure 6D).

Impressively, we observed a striking effect of Klf5 deacetylation in the substantial reinforcement of FGF signaling, particularly from iCAFs to *Krt4*^+^ luminal cells (Figure 7C), whereas the most remarkable enhancement in TNF signaling emerged from *Krt4*^+^ luminal cells directed toward iCAFs (Figure 7D). These findings support the idea that the FGF-TNF signaling crosstalk enhanced by Klf5 deacetylation mainly occurs between fibroblasts and *Krt4*^+^ luminal cells. Furthermore, trajectory analysis revealed a differentiation pathway from iCAFs to MyCAFs (Figure 7E). In the Klf5^WT^ group, the secretion of Fgf9 occurred when iCAFs were well differentiated. In contrast, in the Klf5^KR^ group, Fgf9 was expressed from the early stages of iCAF differentiation and persisted throughout the course of differentiation (Figure 7E).

### DeAc-KLF5 upregulates CX3CR1 to enhance FGFR1 activation in PTEN-deficient cancer cells.

In addition to the paracrine crosstalk between cancer cells and CAFs, FGF9 was more potent in activating FGFR1 signaling in prostate cancer cells with the *KLF5^KR^* mutant (Figure 5G), suggesting that the overactivated FGFR1 signaling caused by *KLF5^KR^* knockin could be attributed to additional endogenous molecular mechanisms in cancer cells. Moreover, Klf5 deacetylation activated autocrine signaling prominently in *Krt4*^+^ luminal cells, as indicated by CellChat analysis of the scRNA-Seq data (Figure 4D and Supplemental Figure 6B). On the one hand, FGF signaling from *Krt4*^+^ luminal cells to themselves was elevated in *Klf5^KR^* mouse prostates (Figure 4E and Figure 7D). On the other hand, we conducted a comprehensive analysis of the distinct ligands that mediate autocrine signaling within *Krt4*^+^ luminal cells in *Klf5^KR^* mouse prostates using NicheNet, and then we assessed the efficacy of these ligands in activating FGFR1 signaling using gene set variation analysis (GSVA) and found 25 ligands that activated FGFR1 signaling consistently (Supplemental Figure 7A and Supplemental Data Set 4). Validation of these top ligands and their corresponding receptors in the RNA-Seq data revealed that Cx3cr1 was consistently upregulated in the AP and DP of *Klf5^KR^* mouse prostates and listed on the top of the differential gene list (Supplemental Figure 7, B and C).

**Figure 8:**
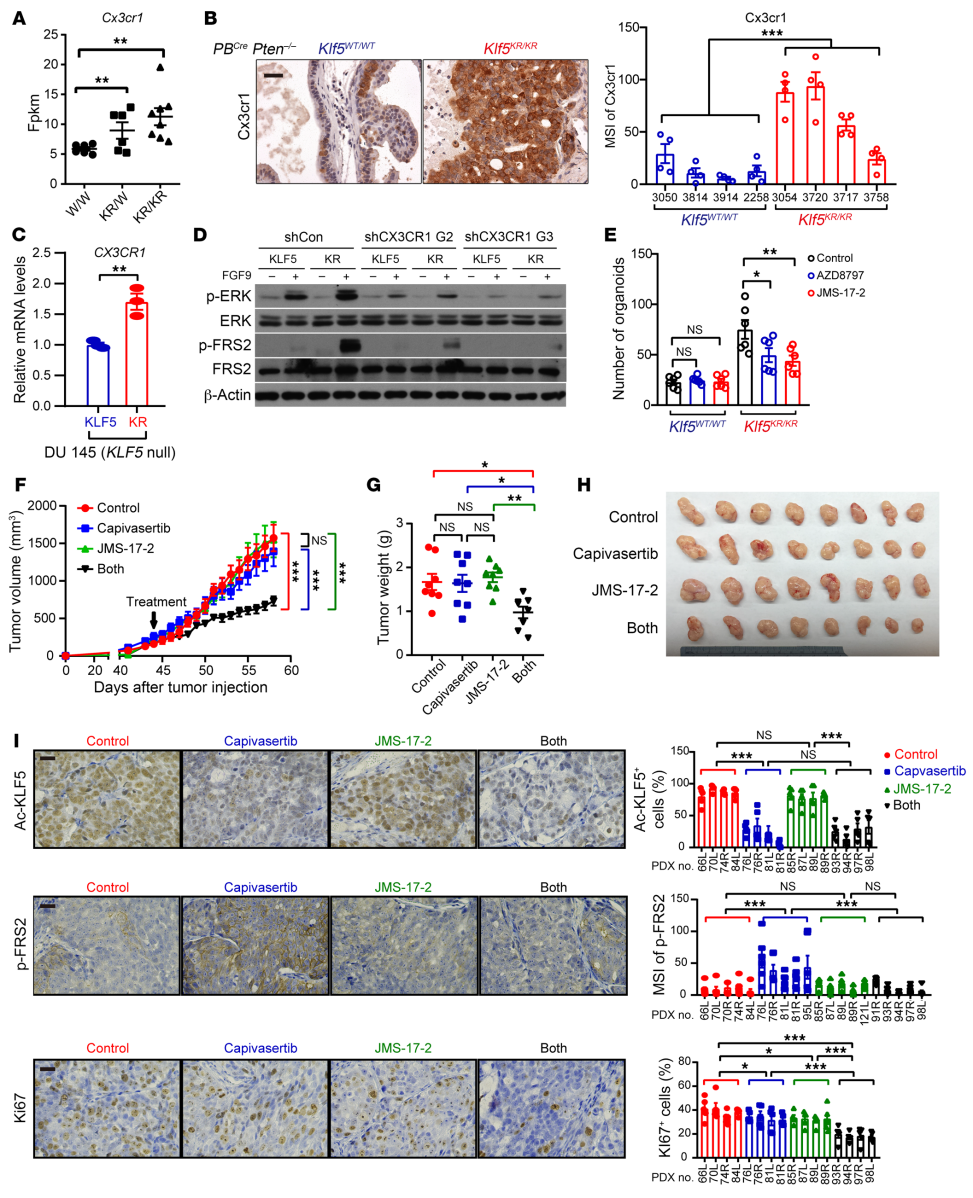
Klf5 deacetylation upregulates CX3CR1 to enhance FGFR1 signaling activity, and blocking CX3CR1 sensitizes tumor cells to AKT inhibition. (**A** and **B**) The expression levels of Cx3cr1 were higher in *PB^Cre^* *Pten^–/–^* *Klf5^KR/KR^* prostate tumors, as indicated by RNA-Seq (**A**) and IHC staining (**B**). *W/W*, *PB^Cre^* *Pten^–/–^* *Klf5^WT/WT^*; *KR/W*, *PB^Cre^* *Pten^–/–^* *Klf5^WT/KR^*; *KR/KR*, *PB^Cre^* *Pten^–/–^* *Klf5^KR/KR^*. Scale bar: 50 μm. (**C**) Expression of *CX3CR1* mRNA in DU 145 prostate cancer cells with KLF5^WT^ (KLF5) or KLF5^KR^ (KR) by real-time qPCR. (**D**) DU 145 cells expressing KLF5^WT^ (KLF5) and KLF5^KR^ (KR) were treated with FGF9 (50 ng/mL) for 5 minutes. FGFR1 downstream p-ERK^Thr202/Tyr204^ and p-FRS2^Tyr436^ were detected by Western blotting. G2 and G3 are 2 shRNAs of CX3CR1. shCon, control shRNA. (**E**) Inhibitors of CX3CR1 selectively suppressed the organoid formation of mouse prostate cancer cells with the *Klf5^KR^* mutant in the context of *Pten* deficiency. AZD8797 (50 nM) and JMS-17-2 (1 nM) are 2 different CX3XR1 inhibitors. (**F**–**H**) *PTEN*-deficient PDXs (The Jackson Laboratory, TM00298) on NSG mice were treated daily with the AKT inhibitor capivasertib and/or the CX3CR1 inhibitor JMS-17-2 as indicated. JMS-17-2 sensitized the effects of capivasertib on PDX growth, as indicated by the tumor volumes at different time points (**F**), tumor weights (**G**), and images (**H**) at excision. (**I**) The expression levels of Ac-KLF5, p-FRS2, and Ki67 were evaluated by IHC staining and quantitative analysis. Scale bars: 50 µm. *P < 0.05, **P < 0.01, and ***P < 0.001, by 2-tailed Student’s *t* test (**A**, **C**, **E**, and **G**) and 2-way ANOVA (**B**, **F** and **I**).

The expression level of Cx3cr1 was increased by *Klf5^KR^* knockin in *Pten*-deficient prostate cancer, as suggested by RNA-Seq (Figure 8A) and confirmed by IHC staining of prostate tissues from *PB^Cre^* *Pten^–/–^* *Klf5^KR/KR^* and *PB^Cre^* *Pten^–/–^* *Klf5^+/+^* mice (Figure 8B). Consistently, DU 145 prostate cancer cells with KLF5^KR^ also had increased CX3CR1 expression (Figure 8C). Functionally, knockdown of CX3CR1 suppressed the activation of FGFR1 signaling in DU 145 cells with KLF5^WT^ and KLF5^KR^ and attenuated the hyperactivation of FGFR1 signaling in KLF5^KR^-expressing prostate cancer cells (Figure 8D). The organoid assay was further used to evaluate the effects of CX3CR1 inhibitors on prostate cancer progression in vitro. Consistently, *Klf5^KR^* knockin promoted the organoid formation of *Pten*-deficient prostate cancer cells (Figure 8E and Supplemental Figure 8A), validating the experimental system. Given the potential off-target effects, we chose 2 different CX3CR1 inhibitors, AZD8797 and JMS-17-2. The addition of AZD8797 and JMS-17-2 selectively suppressed the growth of organoids with deAc-KLF5 (Figure 8E and Supplemental Figure 8A), indicating that induced Cx3cr1 by *Klf5^KR^* knockin is an essential mechanism by which *Pten*-deficient prostate cancer cells have an advantage in tumor growth.

### CX3CR1 inhibition sensitizes PTEN-deficient prostate cancer to the AKT inhibitor capivasertib.

*PTEN* deficiency is a prevalent molecular event in advanced prostate cancer and promotes cancer progression by activating PI3K/AKT signaling. Therefore, the AKT inhibitor capivasertib is currently being studied in phase III clinical trials for both mCRPC (NCT05348577) and metastatic hormone-sensitive prostate cancer (NCT04493853). Capivasertib treatment resulted in a decrease in p-Smad2/3 and Ac-Klf5 in the prostates of *Pten*-knockout mice (Supplemental Figure 8, B and C). Deacetylation of Klf5 upregulated CX3CR1 (Figure 8, A–D), and CX3CR1 served as a central hub for both paracrine signaling and an endogenous pathway that triggered FGFR1 activation (Figures 4–8 and Supplemental Figure 8C). Therefore, it is likely that AKT inhibition by capivasertib reduced KLF5 acetylation, which in turn upregulated CX3CR1 expression and thus led to an enhanced activation of oncogenic FGFR1 signaling. We therefore used a patient-derived xenograft (PDX) model with *PTEN* deficiency to determine whether inhibition of CX3CR1 could sensitize prostate cancer cells to capivasertib. The PDX used in this study demonstrated poor responsiveness to capivasertib (Figure 8, F–H, and Supplemental Figure 8, D and E), implying the potential activation of an adaptive resistance mechanism. Strikingly, addition of the CX3CR1 inhibitor JMS-17-2 prominently sensitized these PDXs to capivasertib (Figure 8, F–H, and Supplemental Figure 8, D and E). This result conclusively underscores a synergistic effect achieved through the combination of CX3CR1 inhibitors and AKT inhibitors in prostate cancer treatment. Further evaluation of Ac-KLF5, p-FRS2, and Ki67 after treatment with AKT and/or CX3CR1 inhibitors by IHC staining (Figure 8I), we found that a single inhibitor failed to significantly decrease Ki67^+^ cells. In contrast, inhibitors of AKT and CX3CR1 synergistically reduced Ki67^+^ cells, consistent with the effects on tumor growth (Figure 8, F–H). Moreover, inhibition of AKT signaling by capivasertib resulted in a decrease in Ac-KLF5 and an increase in p-FRS2, validating an adaptive resistance caused by capivasertib. Synergistic inhibition of CX3CR1 successfully dampened FRS2 phosphorylation, rendering the tumors sensitive to capivasertib again (Figure 8I).

### Upregulation of FGF9 and CX3CR1 is associated with FGFR1 activation in Pten-deficient human prostate cancer.

Klf5 acetylation induced by *Pten* deficiency constrained Fgfr1 activation by suppressing Fgf9 and Cx3cr1. We therefore further evaluated whether FGF9 and CX3CR1 are associated with FGFR1 activation in *PTEN*-deficient human prostate cancer.

**Figure 9:**
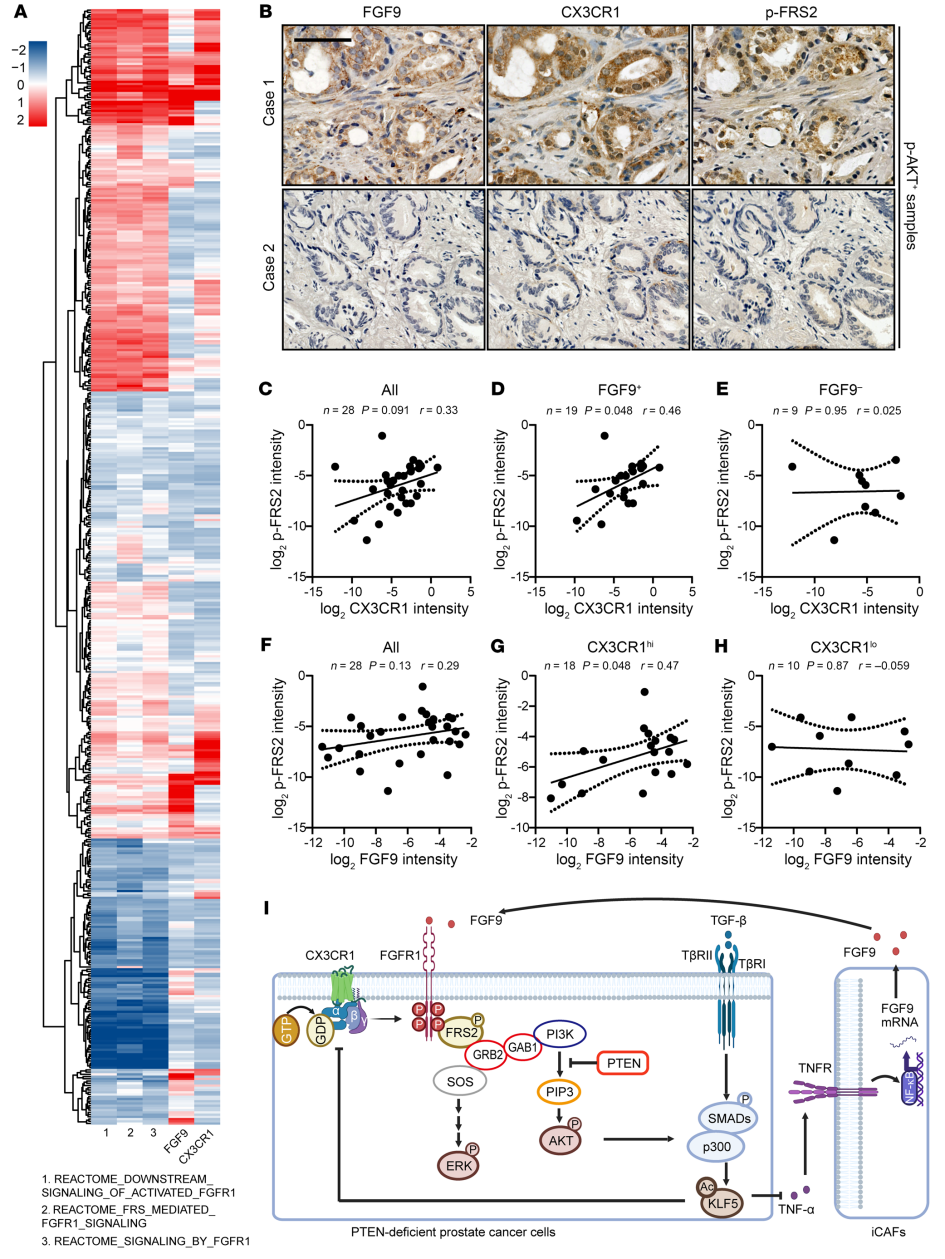
Higher expression levels of FGF9 and CX3CR1 correlate with the activation of FGFR1 signaling in human prostate cancer. (**A**) Correlation of FGF9 and CX3CR1 with FGFR1 activation in prostate cancer samples from TCGA database. ssGSEA was used to identify FGFR1 activation for 499 cancer samples using 3 different REACTOME gene sets. The gene expression levels of FGF9 and CX3CR1 were normalized into a *z* score. (**B**) Representative images of IHC staining of FGF9, CX3CR1, and p-FRS2 in p-AKT^+^ prostate cancer samples. Scale bar: 50 μm. (**C**–**E**) In p-AKT^+^ tumors, the expression levels of CX3CR1 and p-FRS2 were positively correlated in FGF9^+^ conditions. All, all p-AKT^+^ tumors (**C**); FGF9^+^, FGF9^+^/p-AKT^+^ tumors (**D**); FGF9^–^, FGF9^–^/p-AKT^+^ tumors (**E**). (**F**–**H**) In p-AKT^+^ tumors, the expression levels of FGF9 and p-FRS2 are positively correlated in the condition of CX3CR1^hi^. All, all p-AKT^+^ tumors (**F**); CX3CR1^hi^, CX3CR1^hi^/p-AKT^+^ tumors (**G**); CX3CR1^lo^, CX3CR1^lo^/p-AKT^+^ tumors (**H**). The definition of the expression levels of p-AKT, FGF9, and CX3CR1 refer to Supplemental Table 1. *P < 0.05, by Pearson analyses (**C**–**H**). (**I**) Schematic depicting how *PTEN* deficiency–induced KLF5 acetylation constrains prostate cancer progression by attenuating FGFR1 activation via CAF reprogramming. This illustration was generated using BioRender (publication agreement no. CZ26N14CEQ).

We first investigated whether the expression levels of FGF9 and CX3CR1 are associated with FGFR1 activation in TCGA database. To systematically evaluate the activation of FGFR1 signaling, we performed single-sample gene set enrichment analysis (ssGSEA) (33, 34) to identify the levels of FGFR1 activation for 499 cancer samples using 3 different FGFR1-related REACTOME gene sets. Interestingly, both FGF9 and CX3CR1 were positively correlated with the score of FGFR1 activation (Figure 9A and Supplemental Figure 9), no matter which REACTOME gene sets were used to calculate the score in the ssGSEA.

Furthermore, in human prostate cancer tissue assays, we detected p-AKT, FGF9, CX3CR1, and p-FRS2 with IHC staining. Activation of AKT provides a sensitive and reliable evaluation of *PTEN* deficiency (6). We further focused on p-AKT^+^ samples to determine whether FGF9 is an active ligand of FGFR1 and whether CX3CR1 is required for FGFR1 activation in *PTEN*-deficient prostate cancer (Figure 9B and Supplemental Table 1). The canonical substrate of FGFR1, p-FRS2, was used as a marker of FGFR1 activation.

In 28 p-AKT^+^ samples, although higher CXC3CR1 expression was associated with higher p-FRS2 expression, the correlation did not reach significance (Figure 9C). Interestingly, when we categorized the samples with FGF9, we found a significant positive correlation between CX3CR1 and p-FRS2 in FGF9^+^ samples (Figure 9D). But in FGF9^–^ samples, the association between CX3CR1 and p-FRS2 disappeared (Figure 9E). On the other hand, FGF9 tended to positively correlate with p-FRS2, but it was not significant (Figure 9F). The positive correlation between FGF9 and p-FRS2 reached significance in CX3CR1^hi^ prostate cancer samples and disappeared in CX3CR1^lo^ samples (Figure 9, G and H). Collectively, FGF9 and CX3CR1 depended on each other to activate FGFR1 in *PTEN*-deficient prostate cancer.

## Discussion

Genetic mutations are the driving force of prostate cancer progression, and *PTEN* inactivation is one of the most important genetic events. Up to 70% of primary prostate tumors show loss or alterations in at least 1 *PTEN* allele (7). Clinically, *PTEN* loss is correlated with unfavorable clinical outcomes, either alone or alongside other biomarkers, aiding in the differentiation between indolent tumors and aggressive prostate cancer (5). In animal models, single knockout of *Pten* leads to PIN, which can progress to high-grade adenocarcinoma following a long latency, with metastasis occurring rarely (6, 10). This suggests that overcoming barriers caused by *Pten* deficiency is essential for continued progression of prostate cancer. Combined inactivation of *Pten* and *p53* in mouse prostates elicits invasive prostate cancer as early as 2 weeks after puberty and is invariably lethal by 7 months of age (7). *PTEN* inactivation also induces TGF-β/BMP signaling, and knockout of *Smad4* overcomes senescence caused by *Pten* deletion and results in invasive, metastatic, and lethal prostate cancers with 100% penetrance (10). Although previous studies documented that p53 and SMAD4 are molecular barriers induced by *PTEN* deficiency, it remains unknown whether PTMs are essential for the progression of *PTEN*-deficient prostate cancer. Our prior findings indicated that KLF5 acetylation at K369 is a crucial event downstream of TGF-β. TGF-β induces KLF5 acetylation in prostate cancer (20, 35), and Ac-KLF5 induced by TGF-β is essential for TGF-β to suppress cell proliferation and tumor growth (20–23). This study further revealed that *Pten* deletion significantly increased Ac-KLF5 expression levels in mouse prostate (Figure 1), in line with the robust activation of TGF-β signaling (10). Moreover, interruption of Klf5 acetylation promoted tumor growth, accelerated cell proliferation, enhanced the formation of tumor organoids, and altered Smad4-knockout–associated genes in *Pten*-deficient prostate cancer (Figure 2 and Supplemental Figure 2D). Therefore, this study indicates that KLF5 acetylation is a barrier to tumor progression boosted by *PTEN* deficiency and provides evidence for a PTM as an essential molecular event induced by *PTEN* inactivation to stall prostate cancer progression.

Disturbance of the microenvironmental crosstalk between fibroblasts and epithelial cells is crucial for prostate cancer progression. In our study, interruption of KLF5 acetylation remodeled the communication between CAFs and prostate cancer cells, emerging as a pivotal factor enabling *PTEN*-deficient prostate cancer to overcome the progression barriers. FGFs released by fibroblasts act on FGF receptors expressed on the surface of epithelial cells, forming paracrine signaling that is well established and regulates diverse cellular processes of prostate epithelial cells (36, 37). This study deciphered paracrine reciprocal communication between *Pten*-deficient prostate cancer cells and iCAFs coordinated by Ac-KLF5. Interruption of Klf5 acetylation in *Pten*-deficient prostate cancer cells signaled iCAFs through TNF-α to promote FGF9 release, which in turn activated FGFR1 signaling in prostate cancer cells (Figures 4–7). Furthermore, deacetylation of Klf5 caused iCAFs to express FGF9 at the early stage of iCAF differentiation (Figure 7E), supporting the role of KLF5 acetylation in iCAF reprogramming. scRNA-Seq analysis indicated that macrophages and neutrophils were additional sources and receivers of TNF signaling, which was amplified by macrophages and neutrophils in *Klf5^KR^* mouse prostates (Supplemental Figure 5, A and B). Further IF staining assay revealed that the expression of TNF-α was also induced in both epithelial cells and CD11b^+^ macrophages (Supplemental Figure 5C). Cx3cr1 was highly expressed in macrophages and has been well recognized for its role in lineage specification and survival of macrophages (38, 39). The expression of *Cx3cr1* was increased in the macrophages of *Klf5^KR^* prostates in scRNA-Seq data. CellChat revealed that the incoming and outgoing strength of macrophages was enhanced in *Klf5^KR^* prostates (Supplemental Figure 3E). Hence, it is conceivable that macrophages might serve as key contributors within the microenvironment that undergo remodeling due to Klf5 acetylation. CX3CR1 in macrophages has been shown to modulate the secretion of proinflammatory cytokines including TNF-α (40), thus it is likely that the increase in TNF-α release in tumor cells was attributed to the higher level of CX3CR1 in Klf5^KR^ prostate cancer cells. It remains elusive how TNF-α stimulates FGF9 secretion in CAFs. NF-κB, a major downstream signaling factor of TNF-α, has potential binding sites for the FGF9 promoter region, as predicted by online-based software OProf. Future studies may examine whether TNF-α stimulates FGF9 release via NF-κB. Targeting this paracrine communication between cancer cells and CAFs would provide an insight into therapeutic strategies for patients with prostate cancer.

Mechanistic studies indicate that KLF5 constrains *PTEN*-deficient tumors by attenuating FGFR1 signaling (Figure 3). The activation of FGFR1 signaling in prostate cancer cells (*Krt4*^+^ luminal cells in scRNA-Seq) with KLF5 deacetylation was suggested by GSEA utilizing RNA-Seq data from both AP and DP samples (Figure 3C), confirmed by the activation of FRS2, ERK, and AKT, three canonical downstream targets of FGFR1 (25) (Figure 3D), and further addressed by scRNA-Seq analysis (Figure 4). In *Pten*-deficient prostate tumors with *Klf5^KR^* mutant, overactivation of Fgfr1 signaling was further supported by increased Fgf9 secretion and upregulated Cx3cr1 expression (Figures 5 and 8). Notably, FGF9 and CX3CR1 depended on each other to activate FGFR1 in *PTEN*-deficient human prostate cancer (Figure 9). On the other hand, inhibition of AKT by capivasertib reduced Ac-KLF5, which in turn induced FGFR1 activation (Figure 8I and Supplemental Figure 8, B and C), rendering an adaptive mechanism of resistance for AKT inhibitors. In prostate cancer, the expression of FGFR1 is observed in approximately 20% of moderately differentiated cases and 40% of poorly differentiated cases (41). Induced activation of FGFR1 leads to invasive adenocarcinoma with 100% penetrance after a 42-week treatment with chemical inducers of dimerization (42), and knockout of FGFR1 in transgenic adenocarcinoma of the mouse prostate (TRAMP) models result in attenuated tumorigenesis (43). In addition, FGFR1 has been identified as one of the three markers to predict indolent prostate cancer (44). Most recently, FGFR1 activation emerged as a crucial factor in regulating phenotypic plasticity during the transition from CRPC to neuroendocrine prostate cancer (NEPC), which is closely associated with metastatic disease (27). Our findings highlight a microenvironmental pathway for FGFR1 activation and provide a rationale for the combined therapy of AKT and FGFR1 inhibitors in prostate cancer treatment.

Previous studies have suggested oncogenic functions of CX3CR1 in prostate cancer, as the expression of CX3CR1 in prostate cancer epithelial cells directs their circulation to the bone (45, 46), and CX3CL1/CX3CR1 enhance the migration and metastasis of prostate cancer cells (47, 48). However, it remains unknown whether and how CX3CR1 affects FGFR1 signaling. The findings in this study revealed that enhanced expression of Cx3cr1 after *Klf5^KR^* knockin in *Pten*-deficient prostate cancer is an endogenous molecular mechanism by which FGFR1 signaling is activated by its paracrine ligand FGF9 (Figure 8). Knockdown of CX3CR1 or blockage of CX3CR1 by different chemical inhibitors (AZD8797 and JMS-17-2) effectively suppressed FGFR1 activation and the formation of prostate cancer organoids (Figure 8). In patients with prostate cancer, high expression levels of CX3CR1 were required for FGF9 to activate FGFR1 signaling (Figure 9D), and CX3CR1 was positively associated with FGFR1 activation under FGF9 secretion (Figure 9G). These findings disclose a crosstalk between FGFR1 and CX3CR1, although the molecular mechanistic details in this crosstalk remain to be defined. We propose that CX3CR1 could directly phosphorylate FGFR1 upon activation by its ligand CX3CL1. Nevertheless, inhibitors of CX3CR1 effectively sensitized *Pten*-deficient PDXs to the AKT inhibitor capivasertib (Figure 8, F–H).

Our previous studies identified KLF5 acetylation at K369 as a PTM downstream of TGF-β. TGF-β induces KLF5 acetylation via SMAD-recruited p300 acetylase (20, 21). In this study, *PTEN* deficiency led to KLF5 acetylation at K369 in humans and K358 in mice (Figure 1). Inhibition of Akt activation by capivasertib attenuated p-Smad2/3 and reduced Ac-Klf5 (Supplemental Figure 8C), suggesting a role of the complex of p-Smad2/3 and p300 acetylase in the induction of Klf5 acetylation. Senescence has been defined as a crucial cellular event that constrains tumor progression caused by *PTEN* inactivation (7, 10). It is plausible that KLF5 acetylation causes the senescence induced by *PTEN* loss. Our previous study reported that Ras inhibits TGF-β–induced KLF5 acetylation and transcriptional complex assembly (49). Interestingly, RAS activation aids prostate cancer in overcoming the barriers imposed by *PTEN* deficiency (11, 50). This corroborates that the removal of KLF5 acetylation is a crucial event in the progression of prostate cancer. In our most recent study, Ac-KLF5 suppressed tumor growth in subcutaneous prostate cancer xenografts but stimulated bone metastatic lesions by promoting osteoclast differentiation (35). Consistently, *Klf5^KR^* knockin in the GEMM further confirmed the suppressive function of KLF5 acetylation in primary tumor growth. We did not observe metastasis in the bone, liver, or lungs of *PB^Cre^* *Klf5^KR/KR^* *Pten^–/–^* mice within 1.5 years, indicating a role of deAc-KLF5 in suppressing tumor motility (35). It is likely that the whole development of prostate cancer requires a rapid shift of KLF5 acetylation, which endows prostate cancer cells with plasticity and adaptation to different microenvironments. By this mechanism, deAc-KLF5 accelerates tumor growth in primary tumors and switches to its acetylated form for metastasis.

In summary (Figure 9I), this study defines Klf5 acetylation at K358 as a *PTEN* deficiency–induced PTM, which constrains prostate cancer growth by attenuating FGFR1 activation. Interruption of Klf5 acetylation, on the one hand, signals iCAFs to release FGF9 via TNF-α; on the other hand, deacetylation of Klf5 induces CX3CR1 expression in prostate cancer cells. Increased FGF9 and upregulated CX3CR1 cooperate to activate FGFR1 signaling, which leads to the progression of *PTEN*-deficient prostate cancer. *PTEN* deficiency is not only prevalent in prostate cancer, as current clinical trials are using p-AKT inhibitors (e.g., capivasertib) combined with abiraterone as a treatment for patients with metastatic prostate cancer. The findings in this study provide a clinical rationale for the combined use of the CX3CR1 inhibitor JMS-17-2 and the p-AKT inhibitor capivasertib in *PTEN*-deficient prostate cancer.

## Methods

### Sex as a biological variable.

This study focuses on prostate cancer, which is found only in men. Therefore, all the mice used in this study were male mice. Results in male mice are clinically relevant to men.

### Mouse strains.

*Klf5^K358R^-*knockin mice were established in our previous study (24) and donated to The Jackson Laboratory with the name Klf5 < LSL-KR > (stock no. 035317). *PB-Cre4*-transgenic (*PB^Cre^*) mice and *Pten*-floxed mice were purchased from the NCI Mouse Models of Human Cancers Consortium (MMHCC, catalog no. : 01XF5) and The Jackson Laboratory (catalog no. 004597), respectively. The GEMM animals were maintained on a C57BL/6 genetic background. These mice were closely monitored and handled at an Emory University Division of Animal Resources (DAR) facility and the animal facility of the Southern University of Science and Technology. The default temperature for housing animals was controllable within a range of 65°F–86°F, ±1°F of the set point year-round, and the relative humidity was controlled within a range of 40%–50% and within 10% of the set point year-round. By default, animal housing areas were on a 12-hour light/12-hour dark cycle.

NSG mice with PDXs were purchased from The Jackson Laboratory (catalog no. TM00298) via iBio Logistics. These mice were housing at a DAR facility at the Southern University of Science and Technology. Capivasertib and JMS-17-2 were diluted in 10% DMSO, 40% PEG300, 5% Tween-80, and 45% saline for the in vivo assay immediately before injection. PDX mice were treated with capivasertib (2.5 mg/kg/day) and/or JMS-17-2 (2.5 mg/kg/day) via intraperitoneal injections.

### Cell lines.

Prostate cancer PC-3 and DU 145 cell lines were obtained from the American Type Culture Collection (ATCC) and propagated according to the manufacturer instructions (23).

### Tissue microarray.

One tissue microarray (no. PRC1021) containing 7 normal/benign samples and 95 cancer samples was purchased from PANTOMICS. Some tissue cores were torn or had a dark, nonspecific background and had to be excluded from the final statistical analyses. The tissue collection protocol was completed under the approval of the ethics committee of each hospital according to the information from PANTOMICS. The pathological features are available in Supplemental Table 2. 

### IF and IHC.

Tissue sections were deparaffinized in xylene, rehydrated in graded ethanol, subjected to antigen retrieval by boiling the slides in a pressure cooker for 3 minutes in a citrate buffer (10 mM trisodium citrate, pH 6.0), and permeabilized with 0.5% (vol/vol) Triton X-100. For IHC staining, slides were treated with 3% H_2_O_2_ for 10 minutes. For both IF and IHC staining, slides were then incubated with 10% goat serum and then with primary antibodies overnight at 4°C. The primary antibodies used for IF and IHC staining are listed in Supplemental Table 4, including the Ac-KLF5 antibody, which was established and reported in our previous study (21, 24, 35).

For IF staining, secondary antibody Alexa Fluor Dyes (Invitrogen, Thermo Fisher Scientific) were used at 37°C for 1 hour, and DAPI staining was then performed in the dark. Fluorescence images were taken with a Leica SP8 confocal microscope at the Integrated Cellular Imaging Core Facility of Emory University.

For IHC staining, EnVision Polymer-HRP secondary antibodies (Dako) were used at room temperature for 1 hour. After the application of DAB-chromogen, tissue sections were stained with hematoxylin, dehydrated, and mounted. IHC-stained images were analyzed to count cells with positive staining and calculate staining intensities by Fiji software.

### Western blotting.

Briefly, RIPA buffer (Santa Cruz Biotechnology, catalog no. sc-364162A) was used to collect protein from the indicated cells and then loaded onto SDS-PAGE gels (Bio-Rad) for Western blotting. The general protocol followed the procedures on Cell Signaling Technology’s website. The primary antibodies used in this study are listed in Supplemental Table 4.

### scRNA-Seq.

The prostates of 16-week-old *PB^Cre^* *Pten^–/–^* *Klf5^KR/KR^* (KR) and *PB^Cre^* *Pten^–/–^* *Klf5^+/+^* (WT) mice were dissected and minced for scRNA-Seq. Two mice per genotype were used. The minced prostate tissue was sent to BerryGenomics for single-cell preparation, library construction, and the next-generation sequencing. Briefly, the single-cell suspension was prepared with 5 mg/mL Collagenase Type II digestion and TrypLE dissociation (both from Thermo Fisher Scientific) and then filtered using 40 μm cell strainers. The cells were washed 3 times with Dulbecco’s PBS (DPBS) (0.04% BSA) and resuscitated to a concentration of 700~1,200 cells/μL (viability >85%). scRNA-Seq libraries were prepared using the Chromium Single Cell 3′ Reagent Kits, version 3 (10x Genomics), according to the manufacturer’s instructions. For gene expression library construction, 50 ng amplified cDNA was fragmented and end repaired, double-size selected with SPRIselect beads, and sequenced on the NovaSeq platform (Illumina) to generate 150 bp paired-end reads.

### Isolation and coculturing of CAFs.

After washing with PBS, *Pten*-deficient mouse prostate cancer tissues were dissected, cut, and minced into small pieces (1–2 mm^3^), digested in 1 mg/mL collagenase I for 30 minutes at 37°C, and seeded into culture flasks with DMEM containing 10% FBS. Fibroblasts grew outwards from the explants and reached 80% confluence after 2 weeks. These CAFs were passaged and cultured for conditioned medium collection and cocultured with pDU 145 cells prostate cancer cells.

CM were collected from subconfluent CAFs grown in DMEM with 5% FBS for 72 hours. DU 145 prostate cancer cells with KLF5^WT^ or KLF5^KR^ were seeded at a density of 5,000 cells in 24-well plates with 10,000 CAFs. Neutralizing antibodies against human TNF-α (SinoBiology, catalog no. 10602-R10N1), mouse tumor necrosis factor receptor 1 (TNFR1) (R&D System, catalog no. MAB430-100), and TNFR2 (SinoBiology, catalog no. 50128-RN204) were used for blocking TNF-α signaling in the cocultures. After 72 hours, CM were collected from the supernatants of the cocultures for ELISA, and the cocultures were collected for RNA isolation. In the cocultures, gene expression levels in mouse CAFs and human prostate cancer cells were detected by real-time qPCR using species-specific primers.

Additional methods can be found in the Supplemental Methods.

### Statistics.

GraphPad Prism version 8.0.1 (GraphPad Software) was used to plot the data and perform statistical analysis. Readings in all experiments are shown as the mean ± SEM. An unpaired, 2-tailed Student *t* test was used to determine the statistical significance of differences between 2 groups, and *P* values of 0.05 or less were considered statistically significant. Two-way ANOVAs were used for the analysis of the differences between the 2 genotypes. In this scenario, data for each genotype included different images from different animals.

### Study approval.

The experiments using GEMMs were approved by both the IACUCs of Emory University (approval no. PROTO201700496) and Southern University of Science and Technology (approval no. SUSTech-JY202202013). The animal experiments performed using PDXs were approved by the IACUC of Southern University of Science and Technology (approval no. SUSTech-JY202202013).

The PDXs used in this study were purchased from The Jackson Laboratory (catalog no. TM00298). For development of the JAX PDX resource, The Jackson Laboratory established a network of collaborating cancer research centers, which are responsible for any necessary IRB authorizations and patient consents to allow their tumor tissue to be used in research.

The tissue microarrays were purchased from US Biomax. Each specimen collected from any clinic was consented to by both the hospital and the individual under approval of the ethics committee of each hospital.

### Data availability.

The data generated or analyzed during the current study are available within the article, supplemental information, and Supporting Data Values file, or from the corresponding authors upon request. The source data underlying the figures and supplemental figures are provided in the Supporting Data Values file. The bulk sequencing data (corresponding to Figure 3, A–C) are accessible through GEO (GEO GSE253523). The fragments per kilobase per million mapped reads (FPKM) and fold changes of genes are listed in Supplemental Data Sets 1 and 2. The scRNA-Seq data (corresponding to Figures 4 and 7) are accessible through GEO accession number GSE262893. The raw scRNA-Seq data are accessible through BioProject number PRJNA1094424 in Sequence Read Archive (SRA) (https://www.ncbi.nlm.nih.gov/bioproject/PRJNA1094424). The significant marker genes in different Seurat clusters are included in Supplemental Data Set 3.

## Author contributions

BZ designed and performed most experiments, analyzed the data, and wrote and finalized the manuscript. ML and FM performed some key animal experiments and some of the mechanistic studies. SX, YL, WW, and QH performed some of the animal and cellular experiments. BZ, XL, and BGB performed bioinformatics analysis. XD, WD, and JJN performed genotyping. AOO and YC provided pathology consultancy. WZ supervised the study. JTD conceived the project, designed and supervised the study, provided overall guidance, and revised and finalized the manuscript. BZ and JD acquired funding. For the co–first authors, the authorship order was assigned on the basis of their contributions to this work.

## Supplementary Material

Supplemental data

Supplemental data sets 1-4

Unedited blot and gel images

Supporting data values

## Version 1 05/23/2024

In-Press Preview

## Version 2 07/15/2024

Electronic publication
