# Supplementary material for: Interruption of KLF5 acetylation promotes PTEN-deficient prostate cancer progression by reprogramming cancer-associated fibroblasts
Source: J Clin Invest. 2024 May 23;134(14):e175949. doi: 10.1172/JCI175949 (PMC11245161; doi:10.1172/JCI175949)
Supplement: Supplemental data [file jci-134-175949-s063.pdf]

Supplemental information

Interruption of KLF5 acetylation promotes *PTEN*-deficient prostate cancer progression by reprogramming cancer-associated fibroblasts

Zhang et al.

I. Supplemental Figures
II. Supplemental Tables
III. Supplemental Datasets
IV. Supplemental Methods

I. Supplemental Figures

Supplemental Figure 1

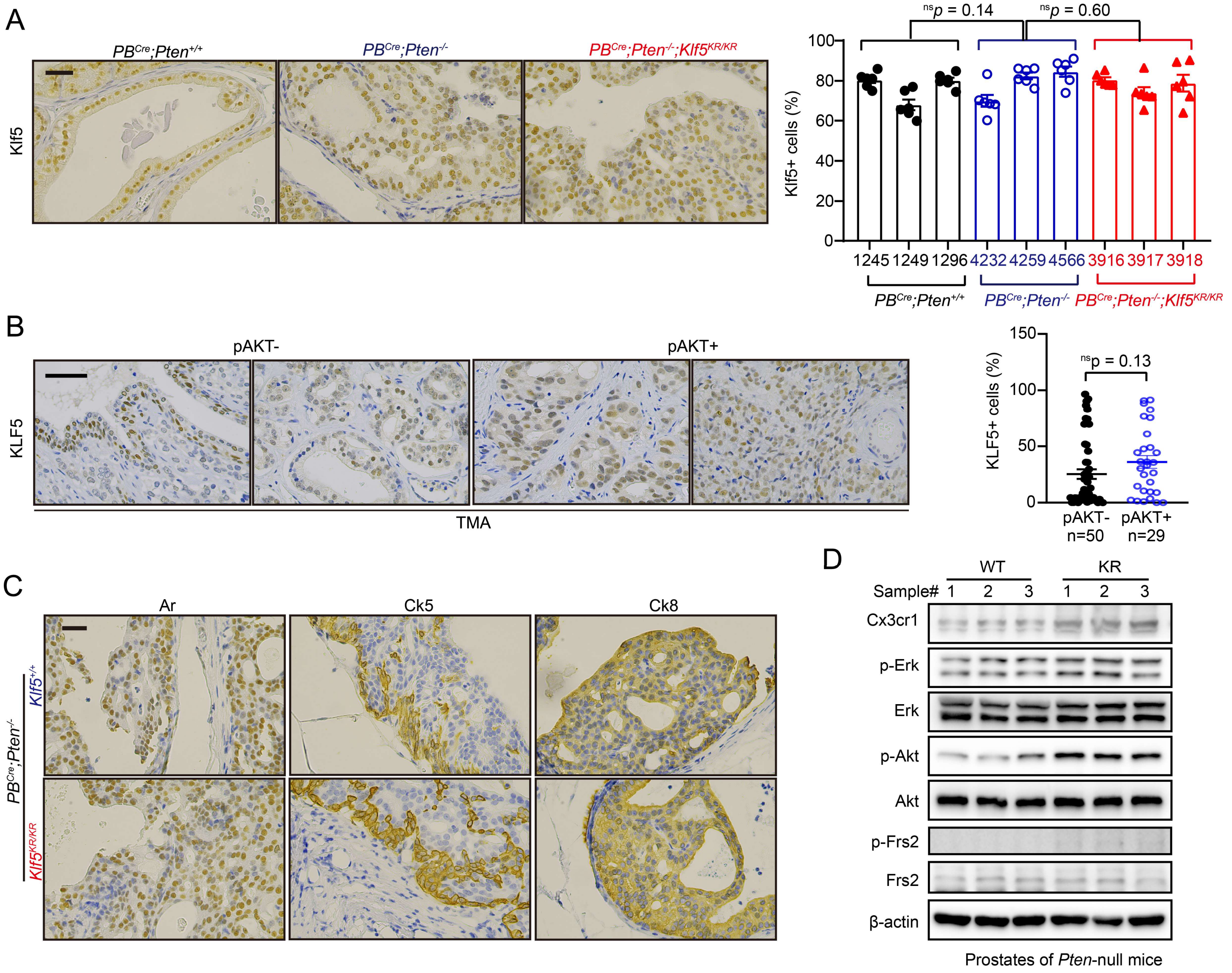

Supplemental Figure 1. *Klf5^{KR}* knockin does not alter the expression levels of total Klf5 but activates Fgfr1 signaling. (**A**) IHC staining of Klf5 in 4-month-old mice with indicated genotypes; (**B**) IHC staining of KLF5 in human prostate cancer specimens with or without AKT activation in TMAs. (**C**) IHC staining of epithelial markers such as Ar, Ck5 and Ck8 with indicated genotypes. (**D**) *Klf5^{KR}* knockin activates Fgfr1 signaling in the context of Pten knockout, as indicated by Western blotting assay. Scale bars, 50 μm.

Supplemental Figure 2

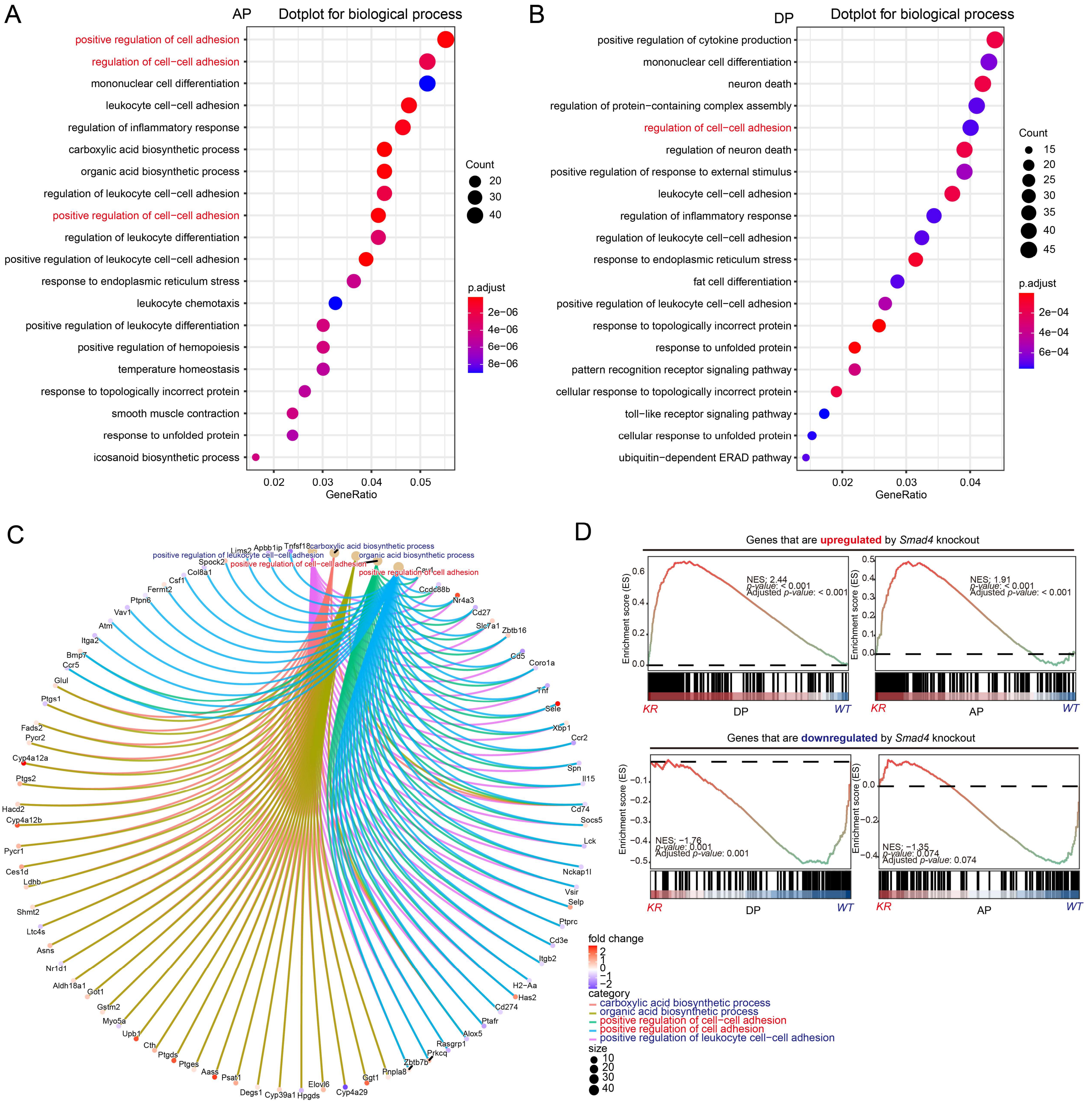

Supplemental Figure 2. Functional annotations of differential gene expression caused by the interruption of Klf5 acetylation in *Pten*-deficient prostate tumor cells. (**A**, **B**) Top 20 significant (adjusted *p*-value < 0.05) Gene Ontology (GO) Biological Process sets in anterior prostates (AP, **A**) and dorsal prostates (DP, **B**). (**C**) Central network plot (CNET plot) for the most significantly enriched GO Biological Process sets and their genes (adjusted p-value < 0.05) associated with deacetylated Klf5 in AP. (**D**) The differentially expressed genes caused by *Klf5*^{KR} knockin were closely associated with the altered genes caused by SMAD4 knockout (*Nature*, 2011), as indicated by GSEA.

Supplemental Figure 3

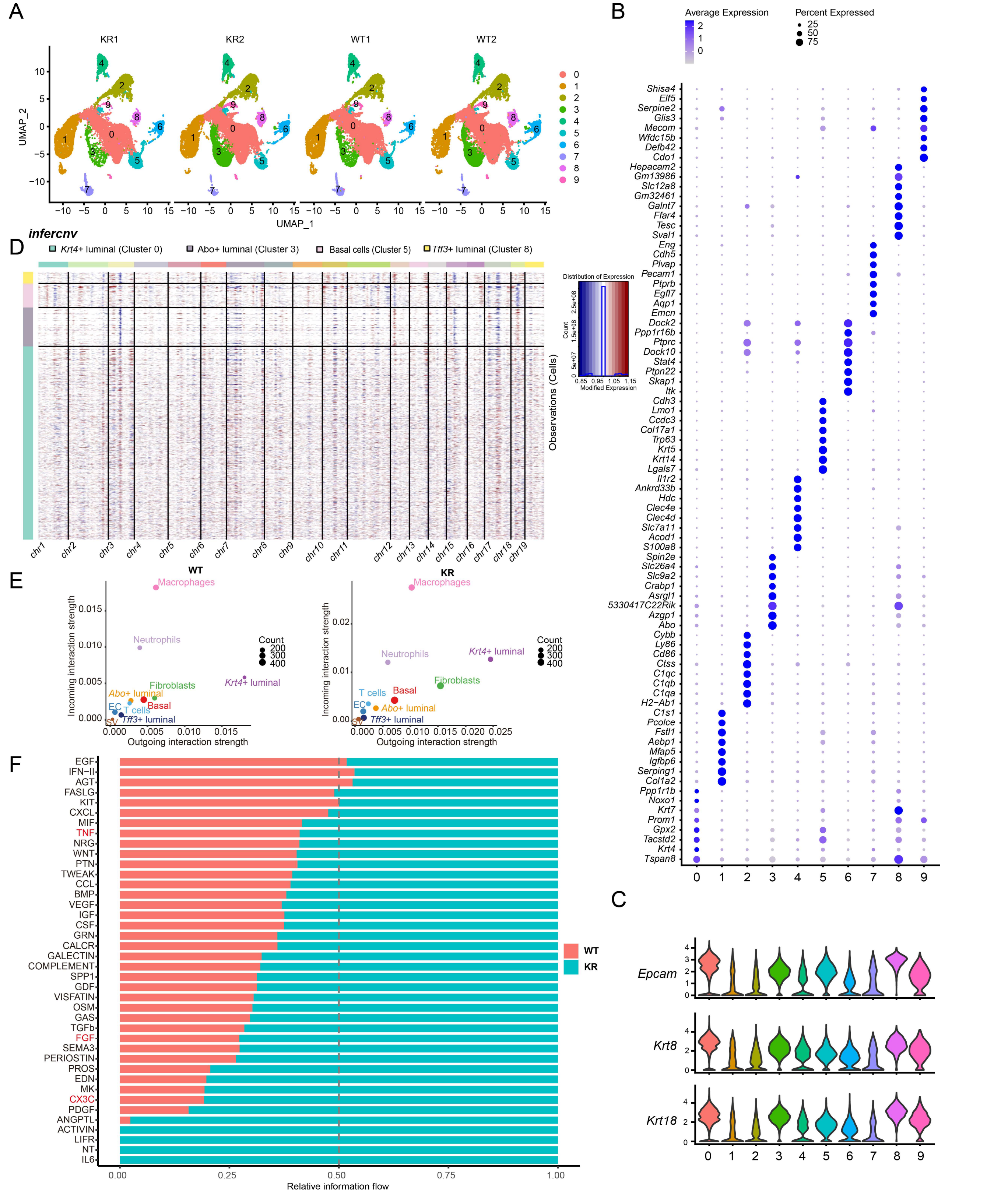

Supplemental Figure 3. Single cell transcriptomic assay clusters different cell types in *Pten*-deficient mouse prostates. (**A**) The distribution of 10 cell clusters in four mouse prostates, as visualized by UMAP. (**B**) Dot plots of the top 10 marker genes of each cluster in the scRNA-seq of mouse prostates (n = 61713 cells). The annotation of each cluster is shown in Fig. 4. (**C**) Representative markers of luminal cells are shown as violin plots. (**D**) *Infercnv* analysis of prostate epithelial cells suggests that the most significant genome variants occur in *Krt4*+ luminal cluster. (**E**, **F**) Cellchat analysis reveals differential strength of interaction (**E**) and top differential microenvironmental signaling (**F**) caused by deacetylation of Klf5.

Supplemental Figure 4

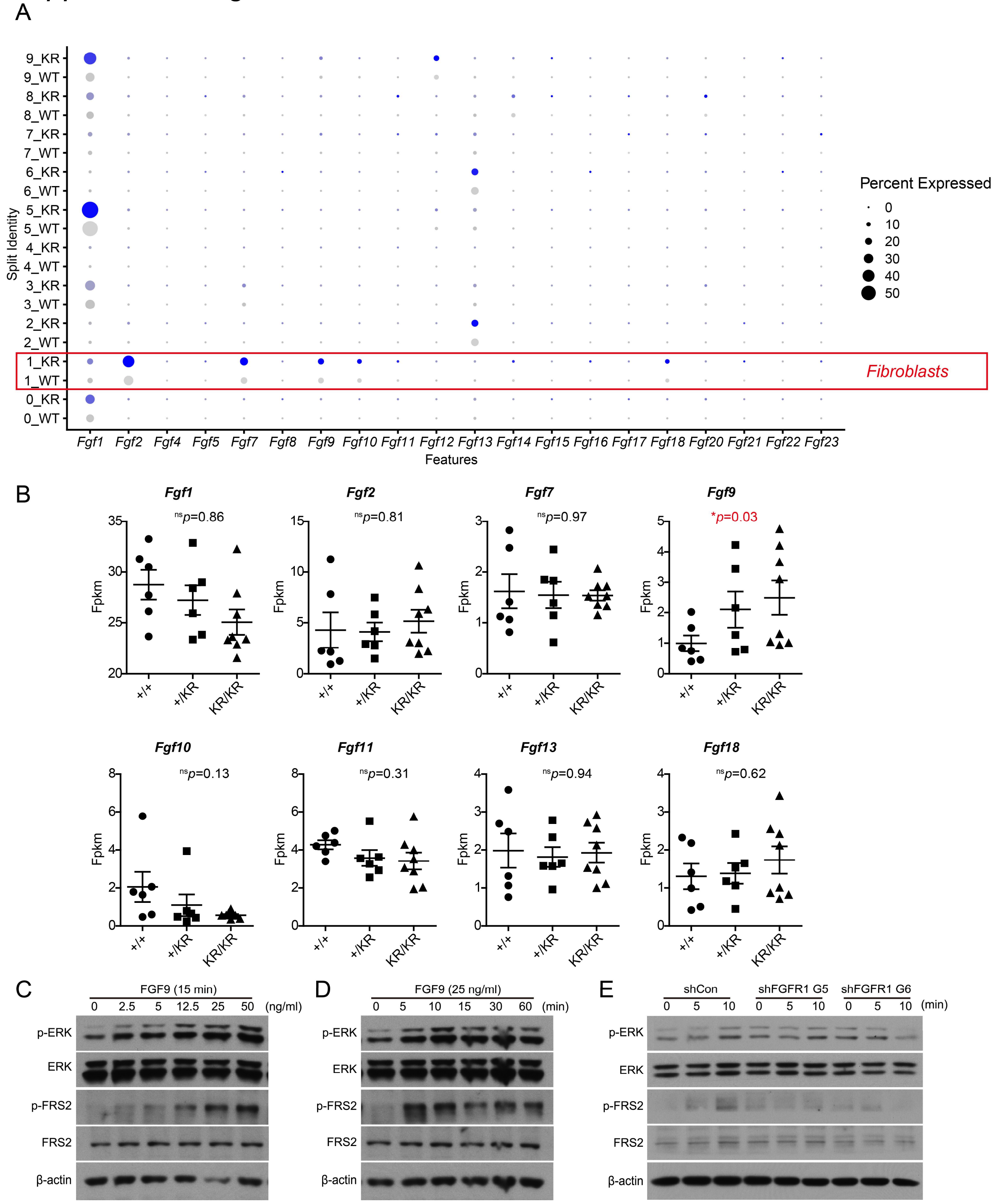

Supplemental Figure 4. Fgf9 in fibroblasts is induced by Klf5 deacetylation in *Pten*-deficient prostate tumor cells and serves as a functional ligand of FGFR1. (**A**) Dot plots of the Fgf expression in different cell clusters of mouse prostates, as indicated by scRNA-seq. Cluster numbers refer to the annotations in Fig. 4A. (**B**) The mRNA levels of Fgfs were determined by RNA-seq in mouse prostates with indicated genotypes. Only genes with Fpkm > 1, at least in one group, are shown. +/+, *PB*^{Cre}*;Pten*$^{-/-}$*;Klf5*$^{WT/WT}$; +/*KR*, *PB*^{Cre}*;Pten*$^{-/-}$*;Klf5*$^{WT/KR}$; *KR/KR*, *PB*^{Cre}*;Pten*$^{-/-}$*;Klf5*$^{KR/KR}$. Data are shown in mean ± S.E.M. (**C**, **D**) FGF9 induced the activation of FGFR1 signaling, as indicated by the Western blotting assay detecting p-ERK$^{Thr202/Tyr204}$ and p-FRS2^{Y436}. DU 145 cells were treated as indicated in a dose curve (**C**) and a time curve (**D**). (**E**) Silence of FGFR1 suppressed the activation of ERK and FRS2, as indicated by Western blotting. G5 and G6 are two different FGFR1 shRNAs. DU 145 cells were treated with FGF9 (25 ng/mL) as indicated time.

Supplemental Figure 5

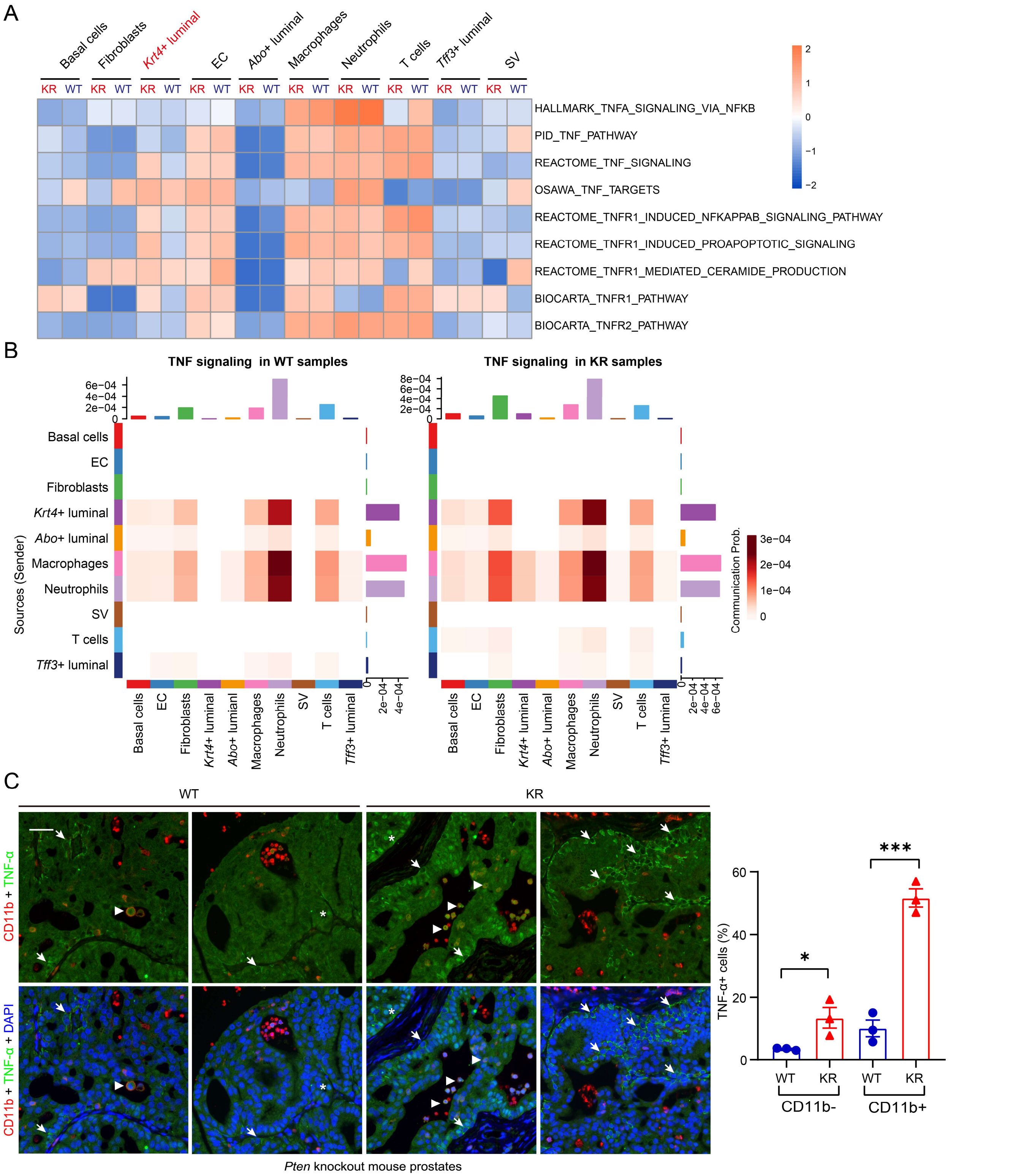

Supplemental Figure 5. Klf5 deacetylation enhances TNF signaling in prostate tumor microenvironment. (**A**) TNF signaling activities of each cell cluster were calculated by Gene Set Variation Analysis (GSVA) and plotted as a heatmap. TNF signaling related datasets were collected from MSigDB. (**B**) Fibroblasts in PB^{Cre};$Pten^{-/-}$;$Klf5^{KR/KR}$ (KR) group receives enhanced TNF signaling from *Krt4*+ luminal, macrophages and neutrophils relative to PB^{Cre};$Pten^{-/-}$;$Klf5^{W/W}$ (WT) group, as indicated by *Cellchat*. (**C**) TNF-α was enhanced by $Klf5^{KR}$ knockin in both epithelial cells and CD11b+ macrophages, as indicated by IF staining. Scale bars, 50 μm. White arrowheads indicate CD11b+/TNF-α+ cells. White arrows indicate CD11b-/TNF-α+ cells. White stars indicate nuclear staining of TNF-α, which was excluded from the statistical analysis of TNF-α+ cells on the right.

Supplemental Figure 6

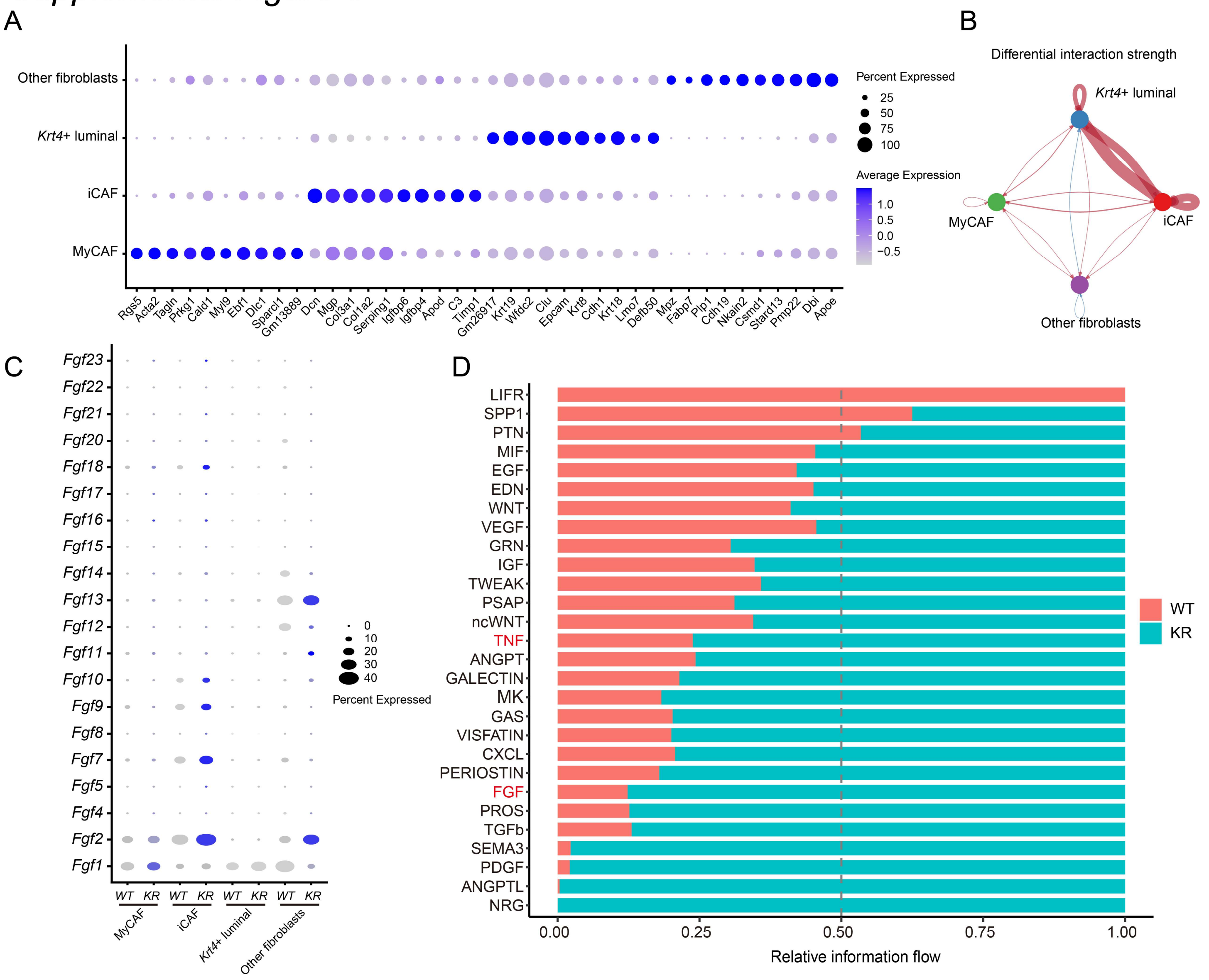

Supplemental Figure 6. Klf5 deacetylation enhances signaling crosstalk between prostate cancer cells and iCAFs. (**A**) Dot plots of top 10 marker genes of *Krt4*+ luminal and different fibroblast clusters in scRNA-seq (n = 35343 cells). (**B**) *KR* knockin enhances the strength of interaction between *Krt4*+ luminal cells and iCAFs. (**C**) Dot plots of Fgfs in *Krt4*+ luminal and different fibroblast clusters. (**D**) Top differential signaling crosstalk between *Krt4*+ luminal and fibroblasts caused by KR knockin. *Cellchat* was used to generate panels **B** and **D**.

Supplemental Figure 7

A

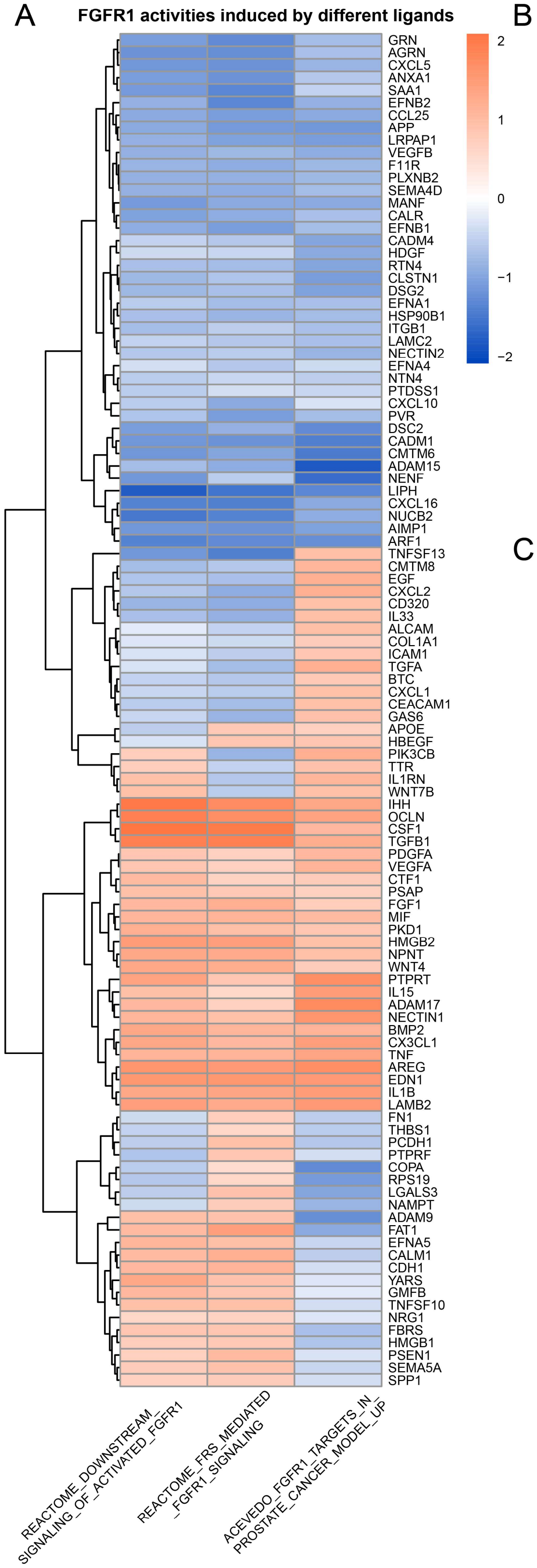

B

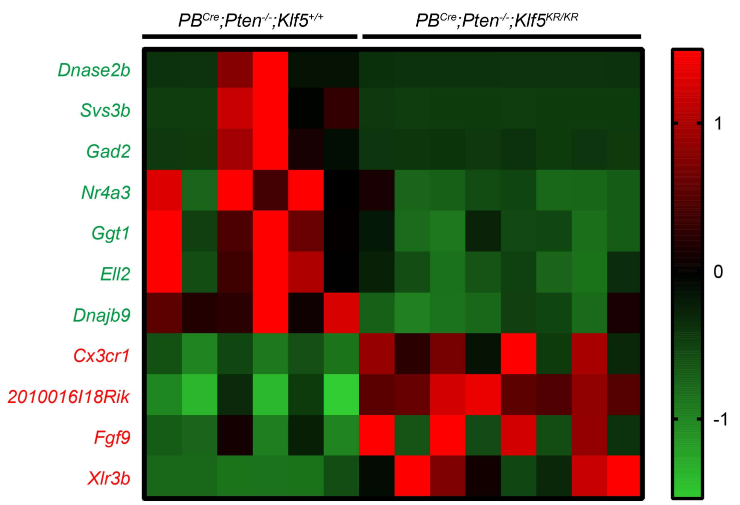

C

ENTREZID	Symbol	Homolog in *H.sapiens*	Function
56629	*Dnase2b*	*DNASE2B*	Hydrolyzes DNA under acidic conditions
329557	*Svs3b*	*NA*	Sperm capacitation
14417	*Gad2*	*GAD2*	Catalyzes the production of GABA
18124	*Nr4a3*	*NR4A3*	Binds to the B1A response-element; Defects in NR4A3 are a cause of Ewing sarcoma (ES)
14598	*Ggt1*	*GGT1*	Glutathione hydrolase activity
192657	*Ell2*	*ELL2*	An elongation factor that can increase the catalytic rate of RNA polymerase II transcription
27362	*Dnajb9*	*DNAJB9*	Acts as a co-chaperone with an Hsp70 protein
13051	*Cx3cr1*	*CX3CR1*	A receptor for the CX3C chemokine fractalkine and mediates both its adhesive and migratory functions
69206	*2010016 I18Rik*	*NA*	NA
14180	*Fgf9*	*FGF9*	Binds to fibroblast growth factor receptors
574437	*Xlr3b*	*NA*	An imprinted candidate for X-linked parent-of-origin effects on cognitive function

Supplemental Figure 7. CX3CR1 induced by deacetylated Klf5 is a potential activator of FGFR1 signaling. (**A**) A heatmap of FGFR1 activities induced by top differential ligands of *Krt4*+ luminal autocrine signaling. *NicheNet* was used to calculate top differential ligands between KR and WT group in scRNA-seq. GSVA was used to evaluate the FGFR1 activities. (**B**, **C**) Consistent differentially expressed genes in anterior prostates (AP) and dorsal prostates (DP). The selected genes are with FDR < 0.05 in one lobe and *p* value < 0.01 in the other lobe. Their gene expression levels are shown in a heatmap (**B**) and their reported functions are summarized in a table (**C**).

Supplemental Figure 8

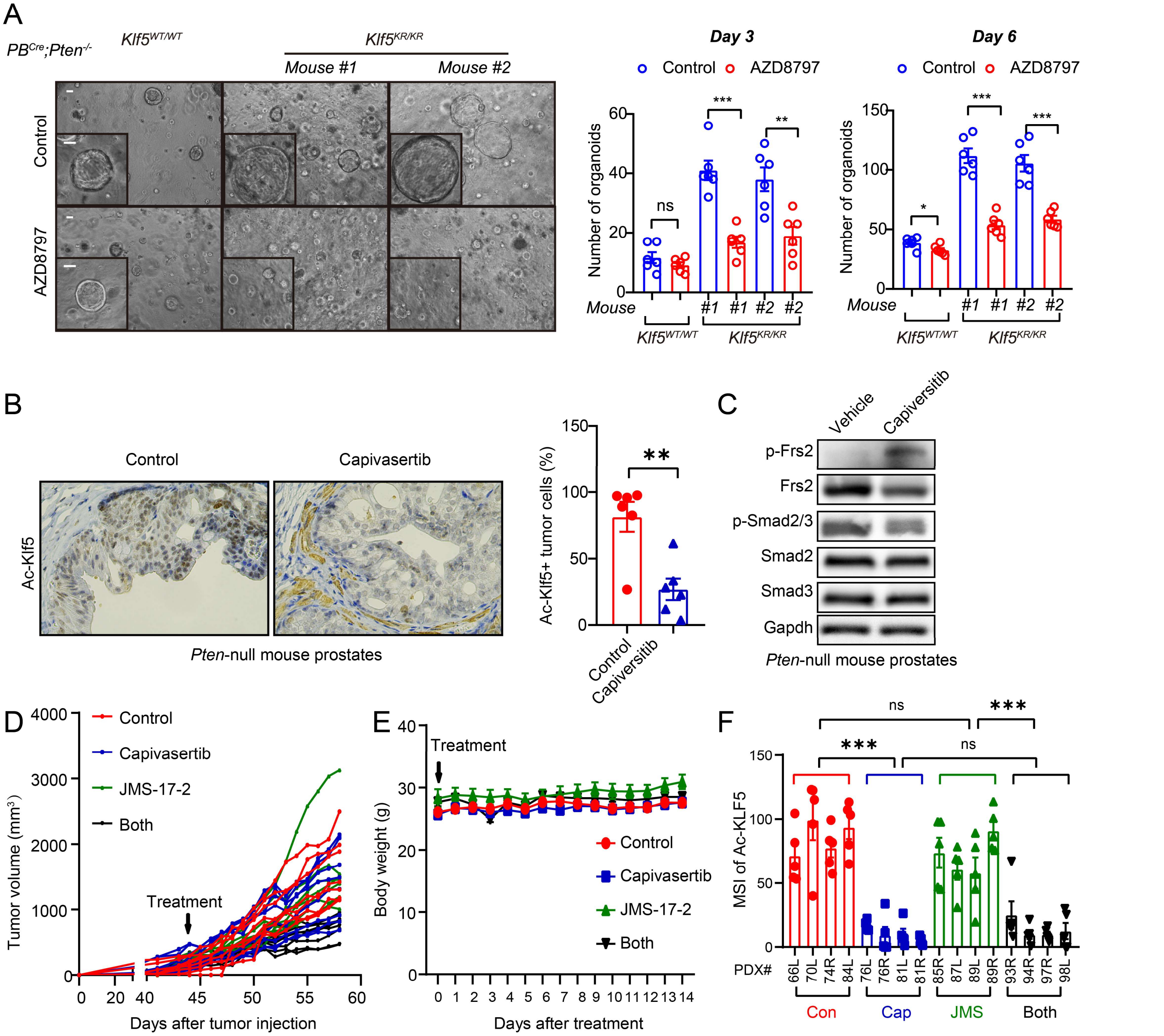

Supplemental Figure 8. CX3CR1 inhibitor suppresses organoid formation and sensitizes PDX to AKT inhibitors. (**A**) CX3CR1 inhibitor AZD8797 (50 nM) selectively suppresses the organoid formation of mouse prostate cancer cells with deAc-KLF5 in the context of *Pten* deficiency. Two-tailed Student's t-tests were performed. ns, not significant; *, $p<0.05$; **, $p<0.01$; ***, $p<0.001$. (**B**, **C**) Treatment of *Pten* knockout GEMM by capivasertib suppressed TGF-β signaling, reduced Ac-Klf5, and enhanced Frs2 phosphorylation, as indicated by IHC staining and Western blotting. (**D**) *PTEN*-deficient PDXs on NSG mice were treated by AKT inhibitor capivasertib and/or CX3CR1 inhibitor JMS-17-2, as indicated in the figures daily. The tumor growth of PDX is shown as tumor volume curve for each PDX on NSG mice. (**E**) Body weight of NSG mice during therapy. (F) The expression levels of Ac-KLF5 were evaluated by quantitative analysis of mean staining intensities (MSI).

Supplemental Figure 9

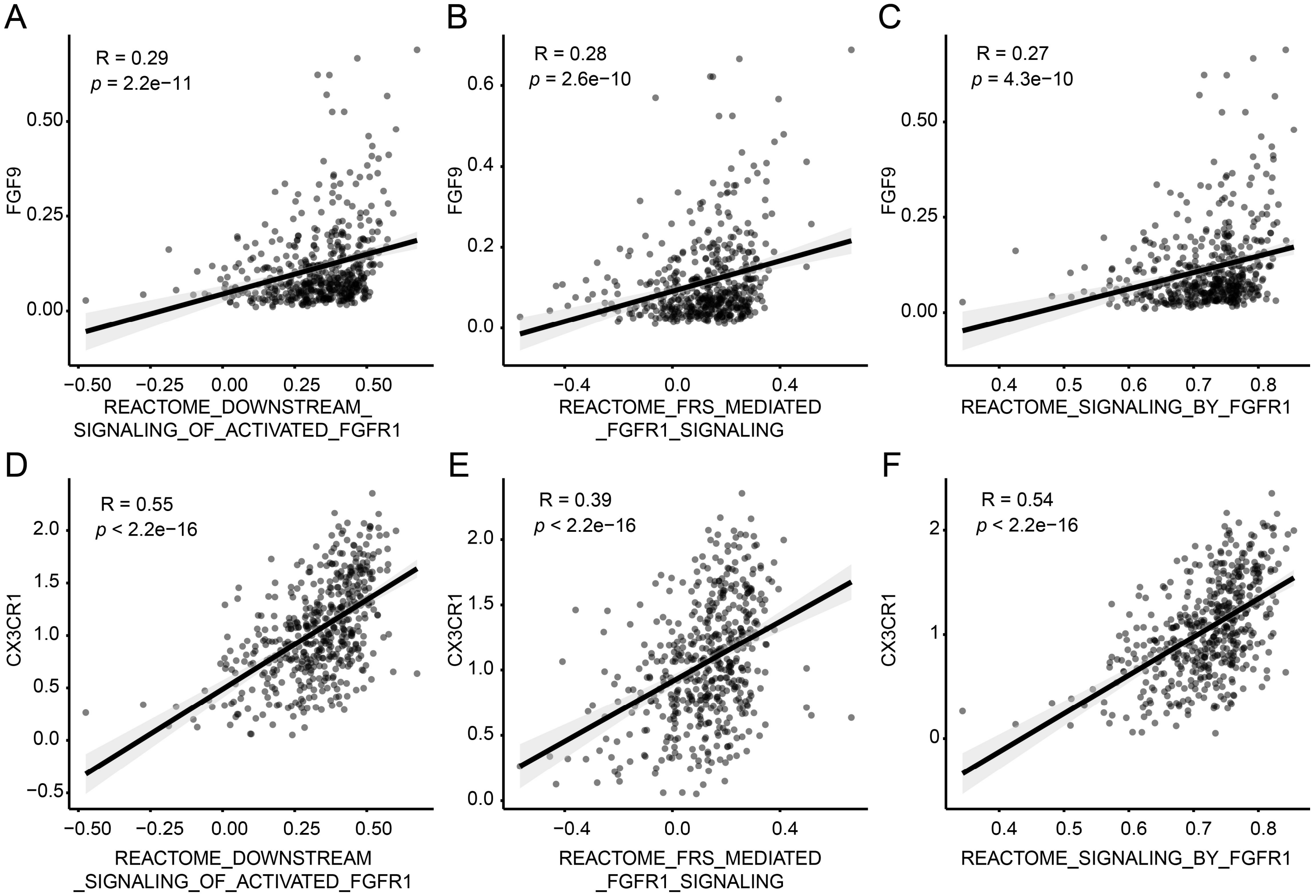

Supplemental Figure 9. Correlation of FGF9 and CX3CR1 with FGFR1 activation in prostate cancer samples from TCGA database. (**A**-**C**) Correlation of FGF9 and FGFR1 activation. (**D**-**F**) Correlation of CX3CR1 and FGFR1 activation. Single-sample geneset enrichment assay (ssGSEA) was used to identify the FGFR1 activation for 499 cancer samples by using three different REACTOME genesets. The gene expression levels of FGF9 and CX3CR1 were normalized into a z-score. Pearson analyses were performed in panels **A**-**F**.

II. Supplemental Tables

Supplemental Table 1. Pathological features of p-AKT+ prostate cancer samples.

ID	Grade	Stage	CX3CR1	FGF9	p-FRS2	CX3CR1	FGF9	p-FRS2
			IHC score			Average IHC intensity		
**A10**	I	T2N0M0	1	2	1	0.160	0.012	0.059
**A13**	II	T2N0M0	2	0	0	0.019	0.002	0.032
**B02**	III	T2N0M0	0	0	0	0.004	0.007	0.000
**B03**	III	T3N0M0	1	0	1	0.291	0.000	0.008
**B04**	II	T2N0M0	1	0	1	0.029	0.003	0.016
**B06**	III	T2N0M0	0	1	1	0.023	0.150	0.009
**B07**	II~III	T2N0M0	3	2	0	0.091	0.046	0.031
**B08**	II~III	T1N0M0	1	3	0	0.037	0.126	0.022
**B10**	I	T2N0M0	3	1	2	0.355	0.035	0.071
**C03**	III	T1N0M0	2	0	0	0.028	0.000	0.004
**C06**	III	T2N0M0	0	0	1	0.000	0.001	0.058
**C07**	III	T2N0M0	2	1	4	0.014	0.030	0.478
**D04**	II	T2N0M0	2	2	1	0.059	0.088	0.031
**D05**	III	T2N0M0	1	0	0	0.054	0.011	0.002
**D07**	III	T2N0M0	2	2	0	0.114	0.028	0.005
**D08**	III	T2N0M0	1	1	1	0.010	0.089	0.001
**D10**	I	T4N0M0	2	2	1	0.415	0.195	0.018
**E02**	III	T2N0M0	3	3	0	0.083	0.098	0.011
**E08**	II	T2N0M0	3	1	1	0.172	0.002	0.005
**E10**	II	T2N0M0	3	3	0	0.006	0.048	0.012
**F04**	III	T2N0M0	2	0	1	0.022	0.005	0.022
**F07**	I	T2N0M0	2	0	1	0.211	0.029	0.091
**F08**	II	T1N0M0	3	2	3	0.350	0.048	0.051
**G01**	II	T2N0M0	2	2	3	0.075	0.001	0.007
**G12**	III	T4N0M0	2	3	3	0.172	0.040	0.041
**H02**	II	T2N0M0	0	2	0	0.001	0.002	0.001
**H04**	III	T2N0M0	4	3	0	1.783	0.110	0.054
**H11**	III	T2N0M0	3	2	1	0.419	0.094	0.060

Note:

The expression levels of CX3CR1, TNF, FGF9, and p-FRS2 were determined by IHC staining. The below criteria were used for grading the IHC images: “0”, none; “1”, 1–25%; “2”, 26–50%; “3”, 51–75%; and “4”, 76–100% cells stained. Two representative images per sample were used for quantitative analysis, and the average intensity of IHC staining images was calculated by Image J software.

Supplemental Table 2. Clinical parameters of patient samples in tissue microarray PRC1021.

Position	Sex	Age	Anatomic Site	Pathology	Grade	Stage
A06	M	60	Prostate	Adenocarcinoma	II	T2N0M0
A08	M	72	Prostate	Adenocarcinoma	III	T2N0M0
A09	M	63	Prostate	Adenocarcinoma	II	T2N0M0
A10	M	76	Prostate	Adenocarcinoma	I	T2N0M0
A11	M	82	Prostate	Adenocarcinoma	III	T2N0M0
A13	M	77	Prostate	Adenocarcinoma	II	T2N0M0
B01	M	72	Prostate	Adenocarcinoma	III	T2N0M0
B02	M	78	Prostate	Adenocarcinoma	III	T2N0M0
B03	M	74	Prostate	Adenocarcinoma	III	T3N0M0
B04	M	69	Prostate	Adenocarcinoma	II	T2N0M0
B05	M	73	Prostate	Adenocarcinoma	III	T2N0M0
B06	M	73	Prostate	Adenocarcinoma	III	T2N0M0
B07	M	77	Prostate	Adenocarcinoma	II~III	T2N0M0
B08	M	77	Prostate	Adenocarcinoma	II~III	T1N0M0
B09	M	77	Prostate	Adenocarcinoma	III	T2N0M0
B10	M	62	Prostate	Adenocarcinoma	I	T2N0M0
B12	M	67	Prostate	Adenocarcinoma	II	T2N0M0
B13	M	73	Prostate	Adenocarcinoma	II	T2N0M0
C01	M	74	Prostate	Adenocarcinoma	I~II	T2N0M1
C02	M	70	Prostate	Adenocarcinoma	III	T2N0M0
C03	M	66	Prostate	Adenocarcinoma	III	T1N0M0
C04	M	75	Prostate	Adenocarcinoma	III	T2N0M0
C06	M	78	Prostate	Adenocarcinoma	III	T2N0M0
C07	M	79	Prostate	Adenocarcinoma	III	T2N0M0
C08	M	69	Prostate	Adenocarcinoma	I	T1N0M0
C09	M	51	Prostate	Adenocarcinoma	II	T3N1M0
C13	M	54	Prostate	Adenocarcinoma	II	T1N0M0
D01	M	81	Prostate	Adenocarcinoma	III	T2N0M0
D02	M	79	Prostate	Adenocarcinoma	II	T2N0M0
D04	M	77	Prostate	Adenocarcinoma	II	T2N0M0
D05	M	73	Prostate	Adenocarcinoma	III	T2N0M0
D06	M	68	Prostate	Adenocarcinoma	III	T2N0M0
D07	M	78	Prostate	Adenocarcinoma	III	T2N0M0
D08	M	87	Prostate	Adenocarcinoma	III	T2N0M0
D10	M	77	Prostate	Adenocarcinoma	I	T4N0M0
D11	M	85	Prostate	Adenocarcinoma	III	T2N0M0
D12	M	84	Prostate	Adenocarcinoma	III	T2N0M0
D13	M	71	Prostate	Adenocarcinoma	III	T2N0M0
E01	M	73	Prostate	Adenocarcinoma	III	T1N0M0
E02	M	66	Prostate	Adenocarcinoma	III	T2N0M0
E04	M	86	Prostate	Adenocarcinoma	II	T2N0M0
E05	M	75	Prostate	Adenocarcinoma	III	T2N0M0
E06	M	75	Prostate	Adenocarcinoma	II	T2N0M0

E07	M	80	Prostate	Adenocarcinoma	III	T2N0M0
E08	M	71	Prostate	Adenocarcinoma	II	T2N0M0
E09	M	52	Prostate	Adenocarcinoma	III	T2N0M0
E10	M	73	Prostate	Adenocarcinoma	II	T2N0M0
E11	M	73	Prostate	Adenocarcinoma	II	T2N0M0
E12	M	76	Prostate	Adenocarcinoma	II	T2N0M0
E13	M	67	Prostate	Adenocarcinoma	II	T2N0M0
F01	M	76	Prostate	Adenocarcinoma	II	T1N0M0
F02	M	60	Prostate	Adenocarcinoma	III	T2N0M0
F03	M	74	Prostate	Adenocarcinoma	I	T2N0M0
F04	M	73	Prostate	Adenocarcinoma	III	T2N0M0
F05	M	83	Prostate	Adenocarcinoma	II	T2N0M0
F07	M	67	Prostate	Adenocarcinoma	I	T2N0M0
F08	M	54	Prostate	Adenocarcinoma	II	T1N0M0
F09	M	68	Prostate	Adenocarcinoma	I	T2N0M0
F10	M	82	Prostate	Adenocarcinoma	II	T1N0M0
F11	M	59	Prostate	Adenocarcinoma	II	T4N0M0
F12	M	67	Prostate	Adenocarcinoma	I	T2N0M0
F13	M	86	Prostate	Adenocarcinoma	III	T4N0M0
G01	M	75	Prostate	Adenocarcinoma	II	T2N0M0
G04	M	80	Prostate	Adenocarcinoma	II	T2N0M0
G05	M	71	Prostate	Adenocarcinoma	III	T3N0M0
G06	M	67	Prostate	Adenocarcinoma	II	T2N0M0
G07	M	64	Prostate	Adenocarcinoma	III	T2N0M0
G08	M	73	Prostate	Adenocarcinoma	III	T2N0M0
G09	M	71	Prostate	Adenocarcinoma	III	T1N0M0
G10	M	66	Prostate	Adenocarcinoma	III	T4N0M0
G12	M	87	Prostate	Adenocarcinoma	III	T4N0M0
H01	M	61	Prostate	Adenocarcinoma	III	T2N0M0
H02	M	86	Prostate	Adenocarcinoma	II	T2N0M0
H04	M	68	Prostate	Adenocarcinoma	III	T2N0M0
H05	M	64	Prostate	Adenocarcinoma	II	T4N1M0
H06	M	78	Prostate	Adenocarcinoma	II	T2N0M0
H07	M	72	Prostate	Adenocarcinoma	II	T2N0M0
H08	M	75	Prostate	Adenocarcinoma	III	T2N0M0
H09	M	80	Prostate	Adenocarcinoma	III	T2N0M0
H10	M	77	Prostate	Adenocarcinoma	III	T2N0M0
H11	M	69	Prostate	Adenocarcinoma	III	T2N0M0
H12	M	60	Prostate	Adenocarcinoma	I	T2N0M0

Note:

Adenocarcinoma samples with intact IHC staining are using in this study and their pathological data are listed.

Supplemental Table 3. Primers used for realtime qPCR.

Primer name	Primer sequences
Mouse-Fgf9-for	ATGGCTCCCTTAGGTGAAGTT
Mouse-Fgf9-rev	TCATTTAGCAACACCGGACTG
Human-TNF-for	GAGGCCAAGCCCTGGTATG
Human-TNF-rev	CGGGCCGATTGATCTCAGC
Human-CX3CR1-for	AGTGTCACCGACATTTACCTCC
Human-CX3CR1-rev	AAGGCGGTAGTGAATTTGCAC
Human-GAPDH-for	GGTGGTCTCCTCTGACTTCAACA
Human-GAPDH-rev	GTTGCTGTAGCCAAATTCGTTGT

Supplemental Table 4. Antibodies used in this study.

ANTIBODIES	SOURCE	CATALOG NUMBER	APPLICATION
Ki67	Abcam	ab15580	IHC: 1/3000
pAKT	Cell Signaling Technology	4060S	WB: 1/1500; IHC: 1/200
pERK	Cell Signaling Technology	4370S	WB: 1/1500; IHC: 1/300
pFRS2	R&D SYSTEMS	AF5126	IHC: 1/50
FGF9	Santa Cruz	sc-8413	IHC: 1/200
pSMAD2/3	ABclonal	AP0548	WB: 1/1500; IHC: 1/200
Ac-KLF5*	/	/	IHC: 1/250
KLF5*	/	/	IHC: 1:1000
Ck5	Abcam	ab52635	IHC: 1/200
Ck8	Abcam	ab53280	IHC: 1/200
CX3CR1	Abcam	ab8020	WB: 1/1500; IHC: 1/100
TNF-a	Proteintech	60291-1-Ig	IF: 1/100; IHC: 1/200
CD11b	Abcam	ab133357	IF: 1/100
DyLight594 Goat anti Rabbit IgG	Abbkine	A23420	IF: 1/500
DyLight488 Goat anti Mouse IgG	Abbkine	A23210	IF: 1/500
AKT	Cell Signaling Technology	4691S	WB: 1/3000
ERK	Cell Signaling Technology	4695S	WB: 1/3000
pFRS2	Cell Signaling Technology	3861S	WB: 1/1000
FRS2	Santa Cruz	sc-17841	WB: 1/500
Smad2	Cell Signaling Technology	5339T	WB: 1/2000
Smad3	Cell Signaling Technology	9523T	WB: 1/2000
GAPDH	Proteintech	60004-1-1g	WB: 1/3000
β-Actin	Proteintech	66009-1-Ig	WB: 1/3000
HRP-conjugated Affinipure Goat Anti-Rabbit IgG(H+L)	Proteintech	SA00001-2	WB: 1/3000
HRP-conjugated Affinipure Goat Anti-Mouse IgG(H+L)	Proteintech	SA00001-1	WB: 1/3000

* Antibodies specific for Ac-KLF5 and KLF5 were established in our previous study(1, 2).

III. Supplemental Datasets

Supplemental Dataset 1. RNA-seq analysis of differentially expressed genes in anterior prostates. WW, $PB^{Cre};Pten^{-/-};Klf5^{WT/WT}$, n=3 mice; KRW, $PB^{Cre};Pten^{-/-};Klf5^{WT/KR}$, n=3 mice; KRKR, $PB^{Cre};Pten^{-/-};Klf5^{KR/KR}$, n=4 mice.

Supplemental Dataset 2. RNA-seq analysis of differentially expressed genes in dorsal prostates. WW, $PB^{Cre};Pten^{-/-};Klf5^{WT/WT}$, n=3 mice; KRW, $PB^{Cre};Pten^{-/-};Klf5^{WT/KR}$, n=3 mice; KRKR, $PB^{Cre};Pten^{-/-};Klf5^{KR/KR}$, n=4 mice.

Supplemental Dataset 3. Significant marker genes in different seurat clusters in scRNA-seq of mouse prostates. Marker genes are filtered as below: adjust p-value < 0.05, average fold change > 1.5, pct.1 > 0.4, differential pct > 0.2.

Supplemental Dataset 4. **Top ligands in *NicheNet* analysis for the autocrine signaing in *Krt4*+ luminal cells.** Differential ligands after KR knockin were analyzed by *NicheNet* for the autocrine signaling of *Krt4*+ luminal cells. Their receptors are summarized and their capabilities in activating FGFR1 signaling was evaluated by GSVA.

IV. Supplemental Methods

Mouse prostate collection and histological processing

Mice with indicated genotypes were euthanized and a mixture of prostate, urethra, bladder and surrounding connective tissues was isolated. The prostate was then dissected from the mixture in PBS and the wet weights of prostates were measured immediately. Prostate tissues were fixed in 10% neutral buffered formalin overnight, transferred to 70% ethanol, embedded in paraffin, sectioned at 5 μm, and stained with hematoxylin and eosin (H&E) at the Research Pathology Core Lab at the Winship Cancer Institute. The H&E staining slides were further evaluated by urologic pathology expert Dr. Adeboye O. Osunkoya from Emory University.

RNA-seq analyses

Anterior and dorsal prostates of PB^{Cre};$Pten^{-/-}$;$Klf5^{KR/KR}$ and PB^{Cre};$Pten^{-/-}$;$Klf5^{+/+}$ mice were dissected freshly for RNA isolation at the Emory Integrated Genomics Core and proceeded to library construction and RNA-Seq at Novogene (Sacramento, CA) using paired-end 150 bp reads on a NovaSeq.

FASTQ files from sequencing were quality controlled and adapter trimmed using FASTQC (v0.11.5) and mapped to MM10 reference genome using the STAR aligner (v2.5.0a) (3). Putative PCR duplicates were marked and removed with SAMtools (v1.7) for downstream analysis (4). Gene expression levels were determined by the number of fragments per kilobase per million reads (FPKM), similar to a previously described procedure (5). Briefly, reads overlapping exonic regions of UCSC MM10 known genes were determined using the 'summarizeOverlaps' function of the 'GenomicAlignments' (v1.20.1) package in the R/Bioconductor (v3.6.1). DEGs were determined using edgeR (v3.26.5) with an FDR ≤0.05 determining significance (6). FPKM and DEGs were provided in Supplemental Dataset 1-2.

Functional annotations of differential gene expression caused by deacetylated Klf5 in *Pten*-deficient prostate cancer were using Gene Ontology (GO) analysis for enriched biological processes. DOSE R package was used for generating central network plot (CNET plot) to indicate the most significantly enriched GO biological processes and their associated genes (7).

scRNA-seq analysis

Cell Ranger toolkit (v6.0.2) were used to perform de-multiplexing and alignment to the mm10 transcriptome. Default parameters were used to trim the adaptor sequence and remove the low quality reads. The raw output data were processed with the Seurat package (v4.3.0) in R software (v4.1.3) for each individual sample. Further quality control were used to filter cells to those with fewer than 20% mitochondrial RNA content, fewer than 5% red blood cell content, more than 200 unique molecular identifiers (UMI) counts, fewer than 10,000 UMI counts and more than 1000 total number of molecules detected within a cell. A total of 61713 cells from four scRNA-seq samples were combined and used for further bioinformatic analysis.

The gene expression was normalized by using "LogNormalize" method with a scale factor of 10,000. The top 3,000 highly variable genes (HVGs) were identified by using a selction method of "vst", centered and scaled before principal component analysis (PCA) based on these HVGs. The batch effects were removed by Harmony (v0.1.1) based on the top 15 PCA components identified.

The clusters was further analyzed by using "FindClusters" function of the Seurat package with a optimized resolusion of 0.07. The identified clusters were visualized on the 2D map produced with the UMAP method. For sub-clustering analysis, we applied a similar procedure including the variable genes identification, dimension reduction, cell integration with Harmony and the clustering identification to the restricted cluster derived from the overall analysis.

To annotate the cell clusters, marker genes of each cluster with high discrimination abilities among the groups were identified with the FindAllMarkers function in Seurat using the default non-parametric Wilcoxon rank sum test. The cell groups were annotated based on the marker genes and the well-known cellular markers from the literatures (8, 9). Detailed information of the marker genes of each cluster was provided in Supplemental dataset 3.

Subsets with luminal cell markers (cluster 0, 3, 8) were further estimated for their single cell copy number variations (CNVs). Single cell CNVs were estimated with the inferCNV packages (v1.16.0) (10). The inferCNV analysis was performed with parameters including "denoise", a value of 0.1 for "cutoff", none group reference, and a "ward.D2" method for hierarchical clustering of cells.

The single-cell pseudotime trajectories of Fibroblast subset were generated with the Monocle2 package (v2.22.0) in R (11). Only genes with the mean expression ≥ 0.02 were used in the trajectory analysis. The

dimention was reduced by using the parameters “DDRTree” as reduction method and 2 as max components. Genes that changed along with the pseudotime were calculated and visualized by using plot_cell_trajectory function, and then colored by the normalized expression levels of these genes.

CellChat (v1.6.1) was used for analyzing cell-cell communications based on the secreted signaling database (CellChat DB) (12). Briefly, normalized gene expression matrices of cell clusters of interest were input into CellChat. The differential interaction numbers and strengths between genotypes were calculated by the compareInteractions function. The heatmap of communication probabilities of a specific signaling was computed by netVisual_heatmap function and reorganized by Graphpad software according to the communication probabilities. Top differential ligands for the microenvironmental crosstalk of particular interest was calculated by NicheNet (v1.1.1) (13).

Real-time qPCR

Cultured cells at indicated conditions were lysed in the Trizol reagent (Invitrogen) for RNA isolation according to the manufacturer’s instructions. The cDNAs for mRNA expression analysis were synthesized from total RNA using RT-PCR kits from Promega (Madison, WI). Realtime qPCR primers are listed in Supplemental Table 3.

Enzyme linked immunosorbent assay (ELISA)

Expression levels of TNF-α and Fgf9 in the conditioned media were detected by ELISA according to their respective manufacturer’s instructions. The supernatants of cell cultures were stored at -80℃ freezer for less than a week. Human TNF alpha ELISA Kit (Thermo Fisher, cat# BMS223HS) was used for detecting TNF-α. Mouse Fgf9 ELISA kit (MYBIOSOURCE, San Diego, CA, cat# MBS2708203) was used for detecting Fgf9.

Organoid culture

Prostate cancer organoid culture was performed following established procedures (14). Briefly, mouse prostate cancer cells were dissected and digested by 5 mg/mL collagenase II (Thermo Fisher, # 17101015) for 1.5 hours at 37℃, digested to single cells by TrypLE (Life Technologies, # 12605-010), and then filtered with a 40 μm cell strainer. Twenty thousand cells were seeded in 40 μL Matrigel (BD, #356231) onto prewarmed 8-well chamber slides. The culture media contained 50x diluted B27, 1.25 mM N-acetylcysteine, 50 ng/mL EGF, 100

ng/mL Noggin, 500 ng/mL R-Spondin, 200 nM A83-01, 1nM DHT and 10 μM Y27632. After 3-6 days of culture, the total number of organoids was counted and analyzed.

Reference

1. Xing C, Fu X, Sun X, Guo P, Li M, and Dong JT. Different expression patterns and functions of acetylated and unacetylated Klf5 in the proliferation and differentiation of prostatic epithelial cells. *PLoS One.* 2013;8(6):e65538.
2. Chen C, Sun X, Ran Q, Wilkinson KD, Murphy TJ, Simons JW, et al. Ubiquitin-proteasome degradation of KLF5 transcription factor in cancer and untransformed epithelial cells. *Oncogene.* 2005;24(20):3319-27.
3. Dobin A, Davis CA, Schlesinger F, Drenkow J, Zaleski C, Jha S, et al. STAR: ultrafast universal RNA-seq aligner. *Bioinformatics.* 2013;29(1):15-21.
4. Li H, Handsaker B, Wysoker A, Fennell T, Ruan J, Homer N, et al. The Sequence Alignment/Map format and SAMtools. *Bioinformatics.* 2009;25(16):2078-9.
5. Barwick BG, Scharer CD, Bally APR, and Boss JM. Plasma cell differentiation is coupled to division-dependent DNA hypomethylation and gene regulation. *Nat Immunol.* 2016;17(10):1216-25.
6. Robinson MD, McCarthy DJ, and Smyth GK. edgeR: a Bioconductor package for differential expression analysis of digital gene expression data. *Bioinformatics.* 2010;26(1):139-40.
7. Yu G, Wang LG, Yan GR, and He QY. DOSE: an R/Bioconductor package for disease ontology semantic and enrichment analysis. *Bioinformatics.* 2015;31(4):608-9.
8. Guo W, Li L, He J, Liu Z, Han M, Li F, et al. Single-cell transcriptomics identifies a distinct luminal progenitor cell type in distal prostate invagination tips. *Nat Genet.* 2020;52(9):908-18.
9. Chan JM, Zaidi S, Love JR, Zhao JL, Setty M, Wadosky KM, et al. Lineage plasticity in prostate cancer depends on JAK/STAT inflammatory signaling. *Science.* 2022;377(6611):1180-91.
10. Patel AP, Tirosh I, Trombetta JJ, Shalek AK, Gillespie SM, Wakimoto H, et al. Single-cell RNA-seq highlights intratumoral heterogeneity in primary glioblastoma. *Science.* 2014;344(6190):1396-401.
11. Trapnell C, Cacchiarelli D, Grimsby J, Pokharel P, Li S, Morse M, et al. The dynamics and regulators of cell fate decisions are revealed by pseudotemporal ordering of single cells. *Nat Biotechnol.* 2014;32(4):381-6.
12. Jin S, Guerrero-Juarez CF, Zhang L, Chang I, Ramos R, Kuan CH, et al. Inference and analysis of cell-cell communication using CellChat. *Nature communications.* 2021;12(1):1088.

13. Browaeys R, Saelens W, and Saeys Y. NicheNet: modeling intercellular communication by linking ligands to target genes. *Nat Methods.* 2020;17(2):159-62.

14. Drost J, Karthaus WR, Gao D, Driehuis E, Sawyers CL, Chen Y, et al. Organoid culture systems for prostate epithelial and cancer tissue. *Nat Protoc.* 2016;11(2):347-58.
