# Supplementary figures and images for: Interruption of KLF5 acetylation promotes PTEN-deficient prostate cancer progression by reprogramming cancer-associated fibroblasts
Source: J Clin Invest. 2024 May 23;134(14):e175949. doi: 10.1172/JCI175949 (PMC11245161; doi:10.1172/JCI175949)

# Raw Data of Blots

Fig. 5A-DU 145

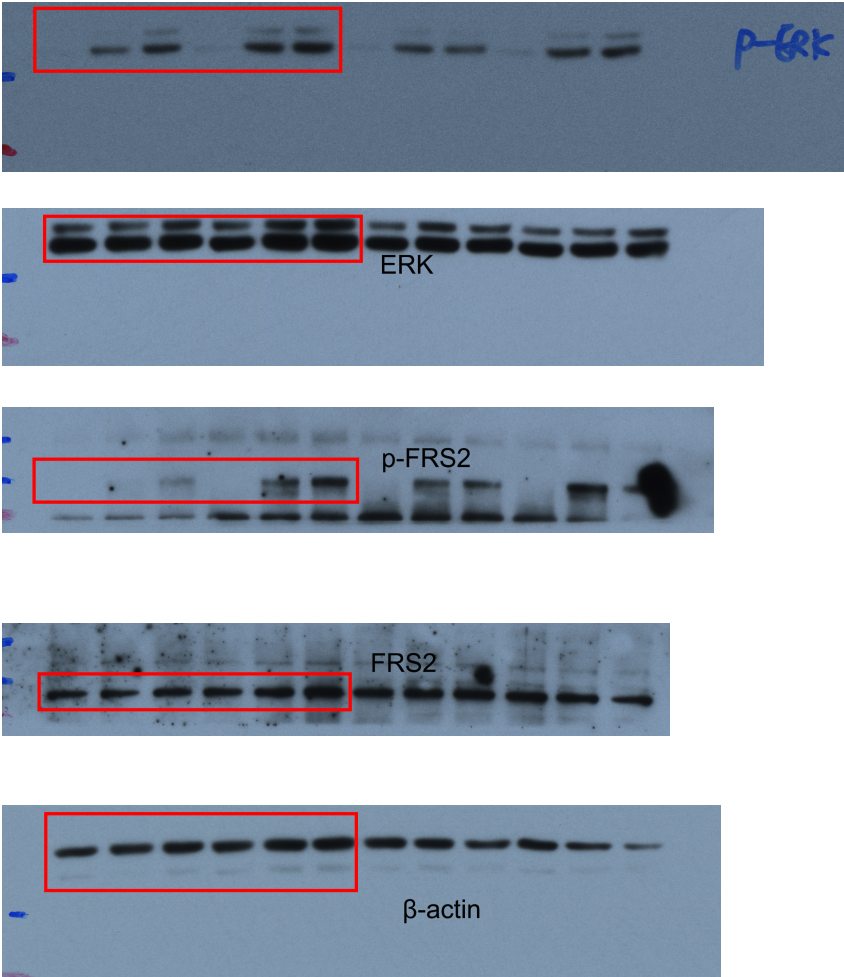

Fig. 5A-PC-3

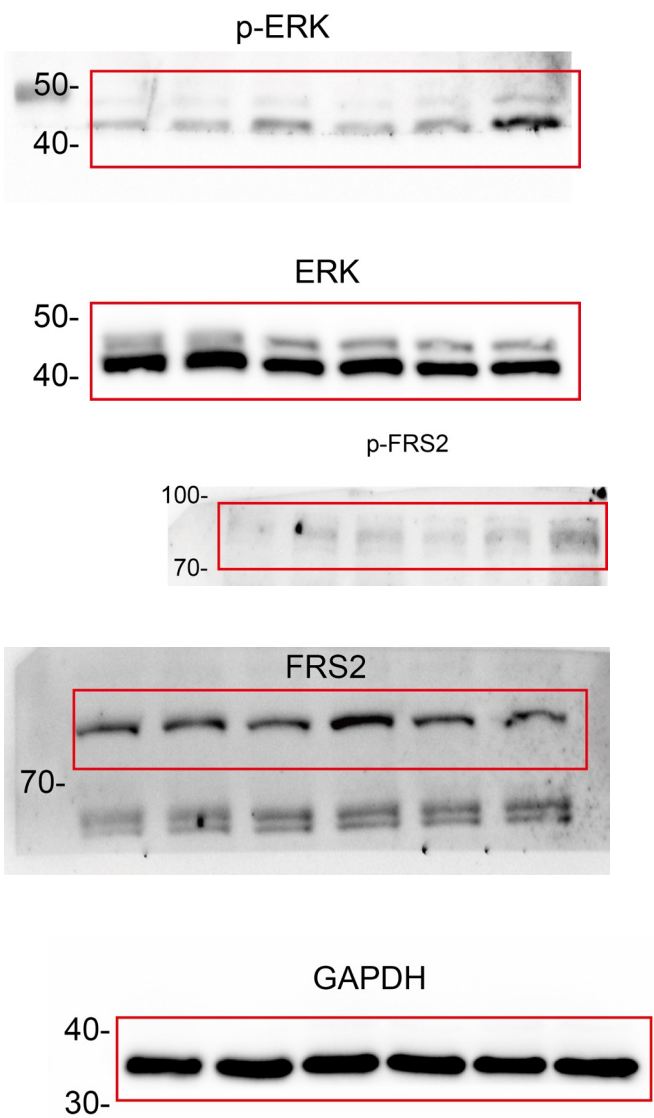

Fig. 5F

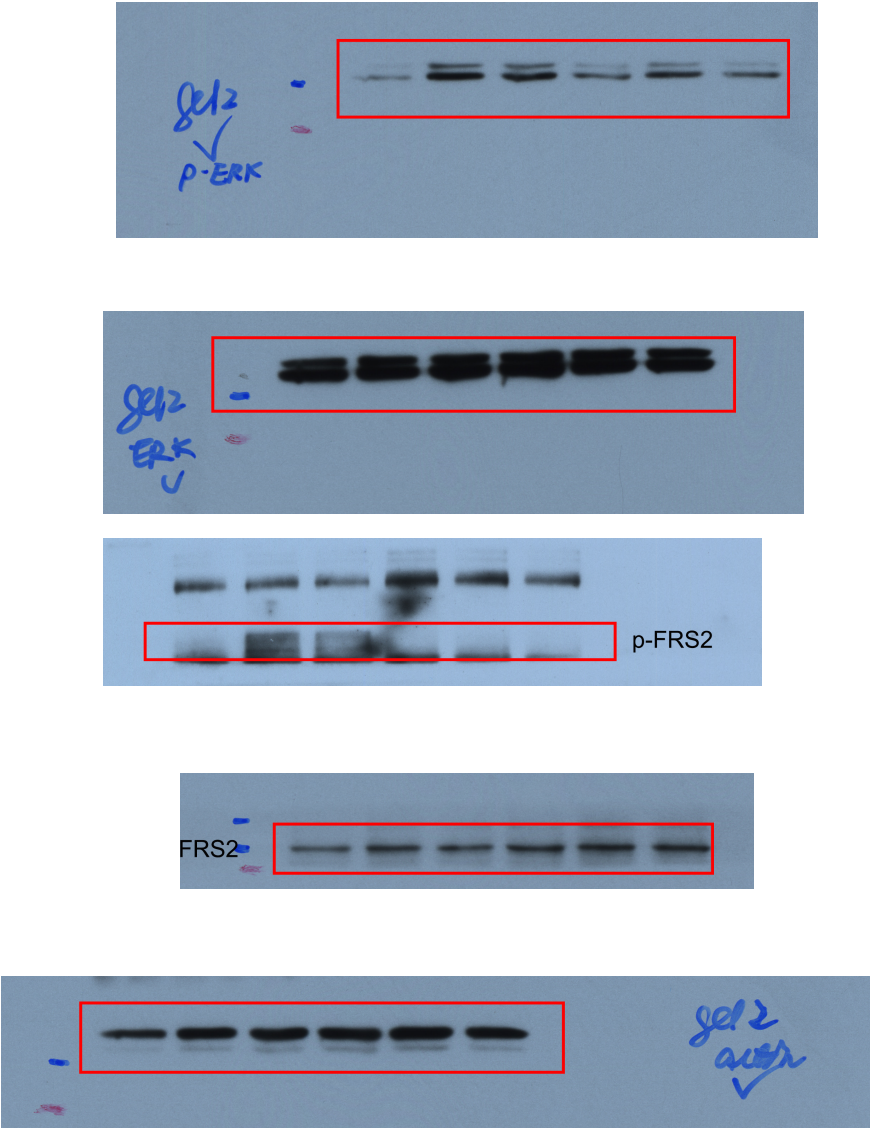

Fig. 5G

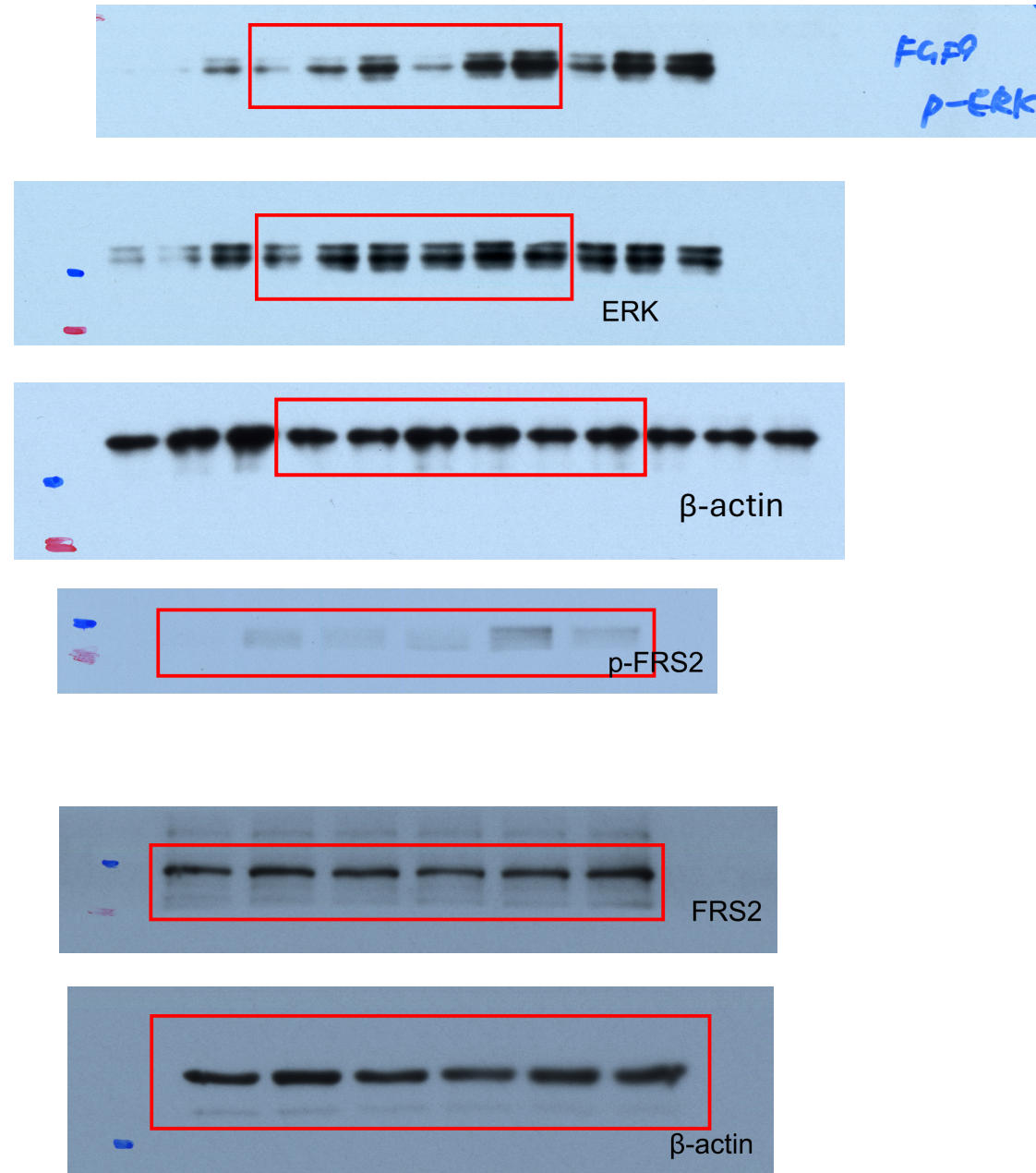

Fig. 8D

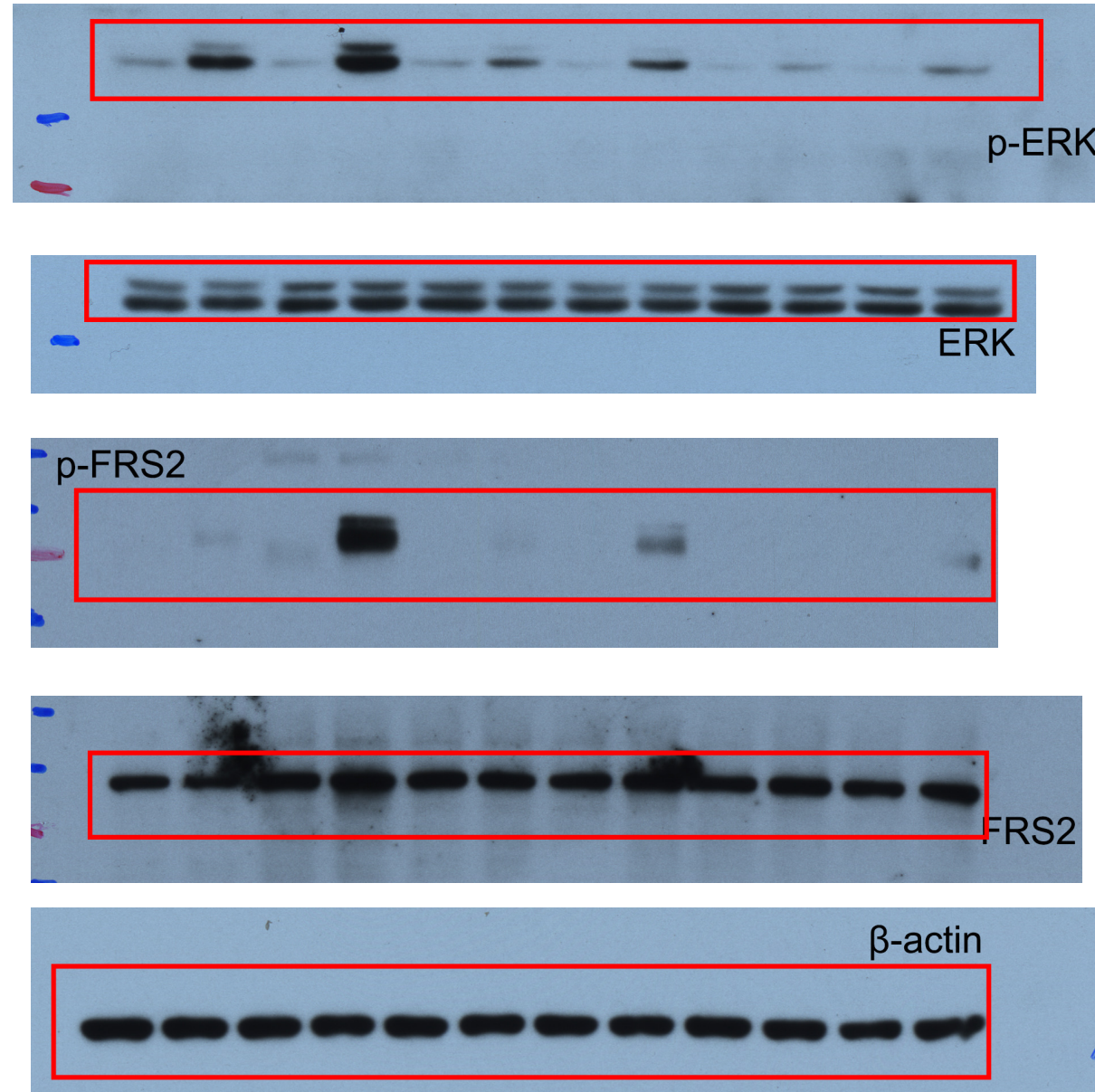

Fig. S1D

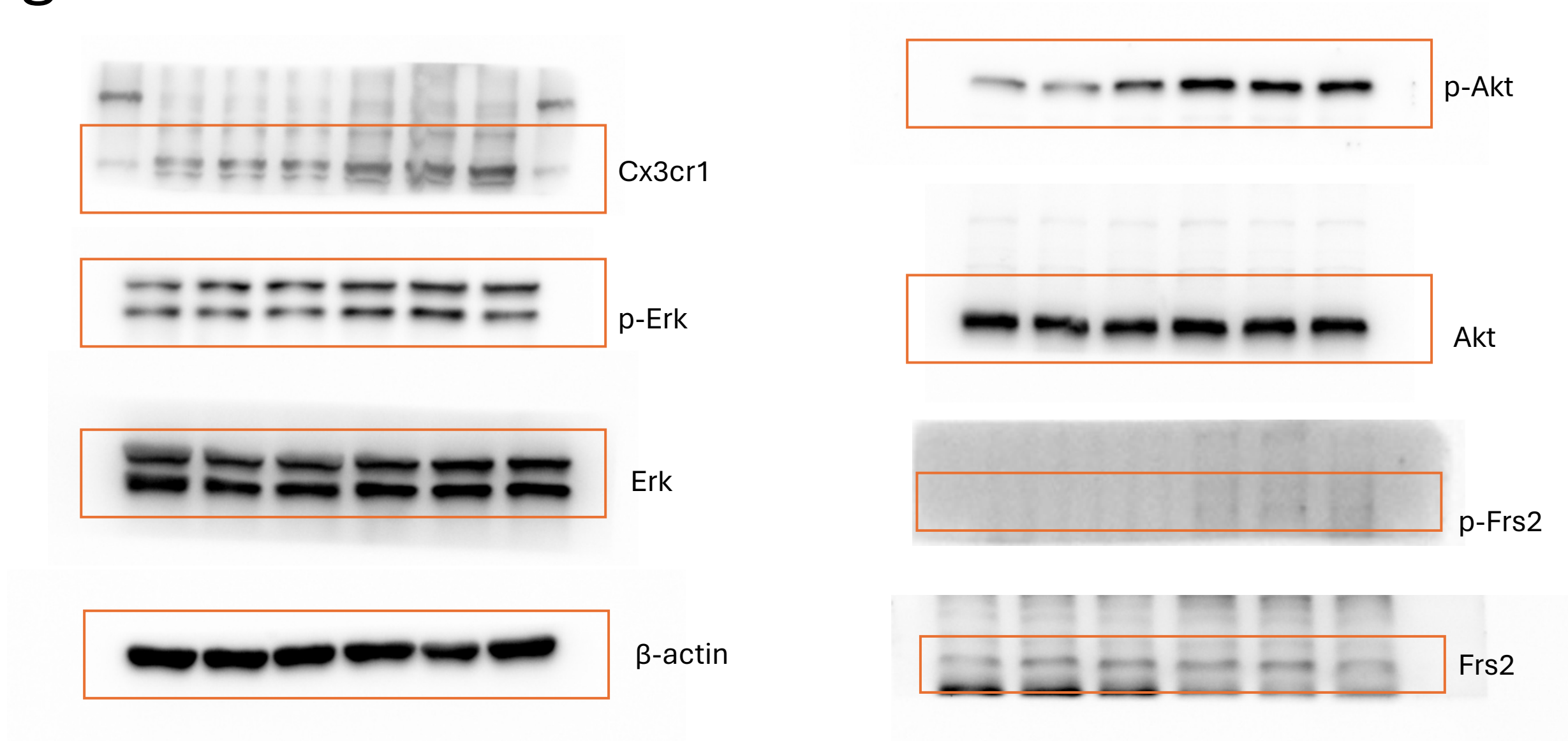

Fig. S4C

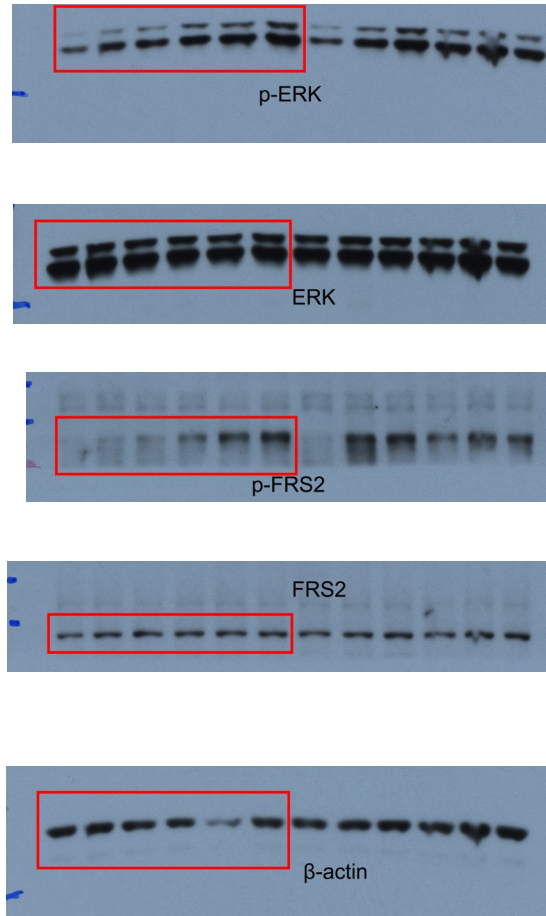

Fig. S4D

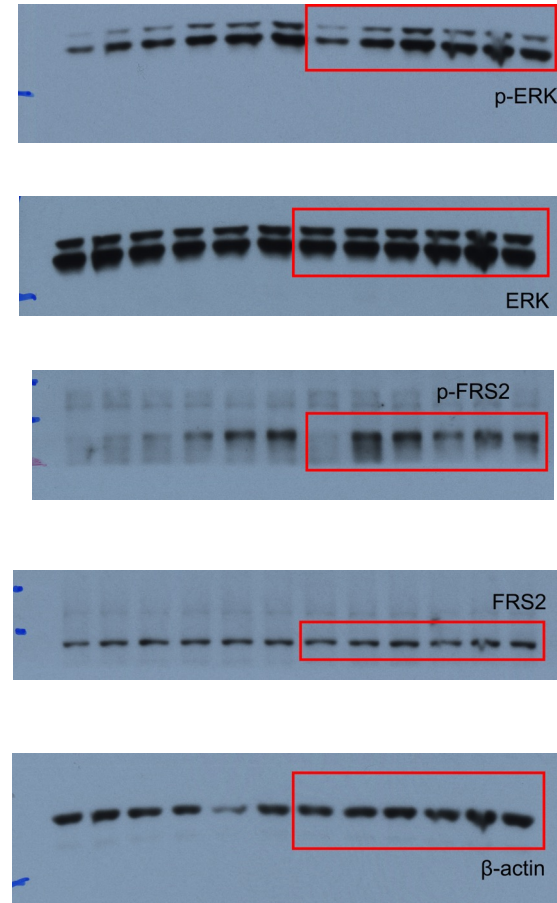

Fig. S4E

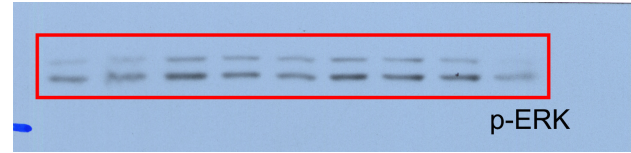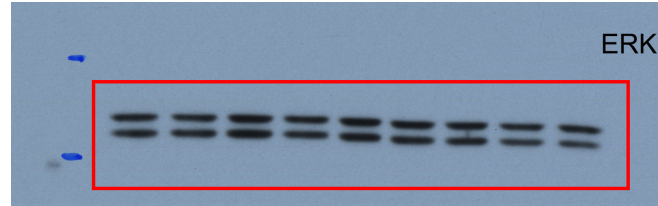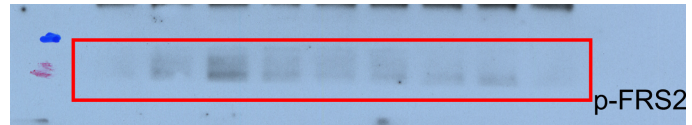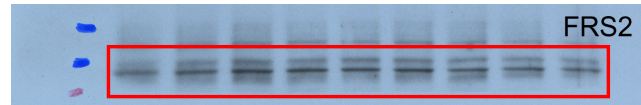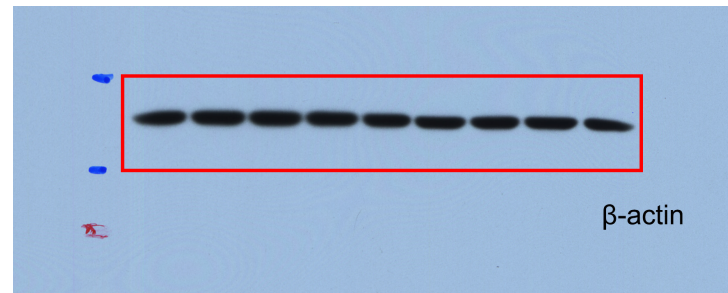

# Fig. S8C

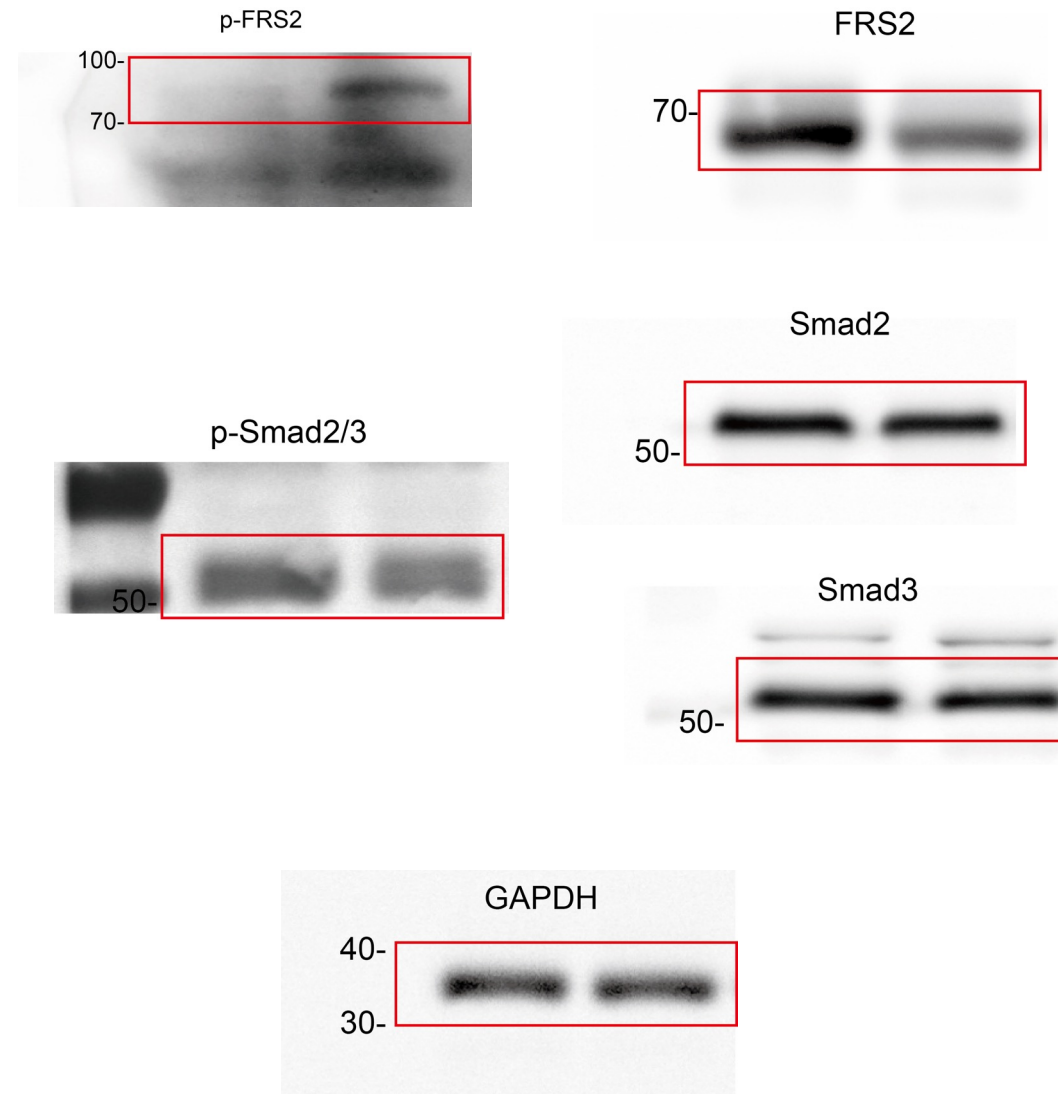

Supplement: Unedited blot and gel images [file jci-134-175949-s065.pdf]
